# Site-Specific Recruitment, Localization of Ionized Monomer to Macromolecular Crowded Droplet Compartments Can Lead to Catalytic Coacervates for Photo-RAFT in Dilution

**DOI:** 10.3390/polym18010106

**Published:** 2025-12-30

**Authors:** Wenjing Niu, Xiyu Wang, Ran Zhang, Yuanli Cai

**Affiliations:** State-Local Joint Engineering Laboratory for Novel Functional Polymer Materials, Jiangsu Key Laboratory of Advanced Functional Polymer Materials, Suzhou Key Laboratory of Macromolecular Design and Precision Synthesis, College of Chemistry, Chemical Engineering and Materials Science, Soochow University, Suzhou 215123, China

**Keywords:** liquid–liquid phase separation, catalytic coacervates, compartmentalization, macromolecular crowding, heterogeneous polymerization

## Abstract

Catalytic coacervates, or droplet reactors, represent a forefront research area in chemistry and materials science. Despite advancements in this field, challenges persist in achieving liquid–liquid phase separation (LLPS) droplet compartmentalization and site-specific reactant recruitment/localization for reaction catalysis, similar to those within biological systems. Herein, we describe the catalytic coacervates for aqueous photo-RAFT in dilution, focusing on the site-specific recruitment/localization of ionized monomer with the aid of *macromolecular crowding and confinement*. Cooperative hydrogen-bonded interpolymer complexation (IPC) of imidazolium-copolymers initiates the ion-cluster formation. Further hierarchical inter-cluster complexation (ICC) leads to the LLPS droplet compartmentalization into charged *dense-phase* and neutral *dilute-phase* compartments. Site-specific recruitment and localization of the oppositely charged monomer into *dense-phase* compartments are achieved by salt-bridging molecular recognition. “Substantial DMA-dilution” (that is, macromolecular crowding) results in sustainable *dense-phase* catalytic sites within *dilute-phase* crowding surroundings, enabling reaction catalysis in dilution (<2% *w*/*w* monomer) to 97% conversion in 12 min. These findings underscore the key roles of *macromolecular crowding and confinement* in the tailorable LLPS droplet compartmentalization and also the site-specific reactant recruitment/localization essential for enzyme reaction catalysis, and provide practical guidelines for creating catalytic coacervates towards lifelike reaction functions.

## 1. Introduction

Complex coacervation [1,2,3,4] of charged proteins and nucleic acids via liquid–liquid phase separation (LLPS) [5] is a key mechanism underlying intracellular organization and compartmentalization [6,7]. The transiently ordered systems exhibit extremely dynamic liquid-like properties [4,8]. As artificial mimics, complex coacervates [9] are a novel class of liquid materials showing low interfacial tension against water [10], semipermeability [11], and living properties [12,13]. Catalytic coacervates [14,15,16], in particular, have been broadly explored for their biosensor [17], nanoreactor [18], and protocell [19] applications. Despite significant advancements [14], achieving lifelike LLPS droplet compartmentalization and site-specific reactant recruitment/localization into the droplet compartments for reaction catalysis under dilute conditions remains a challenge [3,8,14,20].

Polymerization-induced self-assembly (PISA) [21,22,23] including photo-PISA [24,25,26] is robust for scalable synthesis of polymer nanoparticles at high solids contents up to 50% *w*/*w* [27,28]. Charged nanoparticles can be produced via aqueous dispersion RAFT polymerization of ionic liquid monomers [29], and via polymerization-induced electrostatic self-assembly (PIESA) [30,31,32,33] through the polyion complexation of charged growing chains with oppositely charged polyelectrolytes. LLPS-PIESA [34] was exploited for the compartmentalization of dynamic coacervate droplets. However, these coacervates showed a solidification trend, rendering them detrimental for the purposes of catalysis due to the loss of dynamic properties, enabling kinetically-frozen reaction inactivation inside dodecyl-modified polyelectrolyte droplet compartments [35]. In other words, the liquid-like dynamic properties should be maintained during the compartmentalization and reactant recruitment and localization into droplet compartments for sustainable catalytic coacervates [6,14,36].

**Scheme 1:**
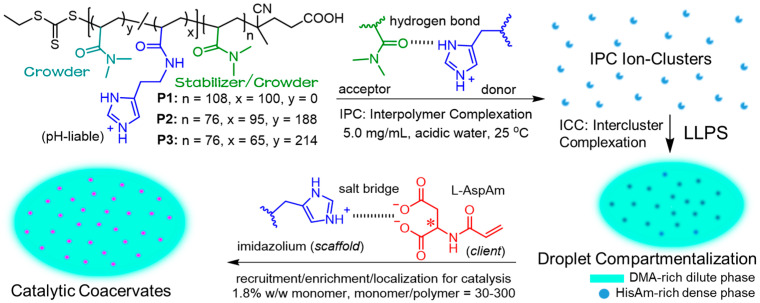
Schematic illustration of catalytic coacervates for aqueous Photo-RAFT in dilution, in which * denotes chiral carbon in L–AspAm monomer.

Biological evidence indicates that *macromolecular crowding and confinement* [37,38,39] are fundamental mechanisms that are closely tied to chemical organization in biological systems [6]. In this work, we aim to explore catalytic coacervates for photo-RAFT in dilution, focusing on site-specific monomer recruitment/localization with the aid of *macromolecular crowding and confinement*. As shown in Scheme 1, we synthesized the ionized histamine acrylamide (HisAm) and nonionic *N*,*N*-dimethylacrylamide (DMA) copolymers. In spite of the water-miscible homopolymers, the block and statistical block copolymers underwent hydrogen-bonded interpolymer complexation (IPC) [40] resulting in complex ion-cluster formation. Hierarchical intercluster complexation (ICC) resulted in pitaya-shaped droplets with cationic HisAm-rich *dense-phase* and neutral DMA-rich *dilute-phase* compartments. These multiphase droplets acted as the scaffolds for site-specific recruitment/localization of anionic L–aspartate acrylamide (L–AspAm) monomer into droplet *dense-phase* compartments via salt-bridging molecular recognition [41,42,43,44]. Furthermore, the “substantial DMA-dilution” (that is, macromolecular crowding [37,38,39]) leads to the sustainable *dense-phase* catalytic sites confined within liquid apolar *dilute-phase* crowding surroundings. Distinct from solvent-miscible macromolecular chain transfer agent (macro-CTA) used for classical PISA formulations, these droplets functioned as the *scaffolds* and *catalytic sites* for the reaction catalysis in <2% *w*/*w* monomer dilution, leading to 97% conversion in 12 min. These active coacervates highlighted a high catalytic efficiency toward lifelike reaction functionalities [45].

## 2. Results and Discussion

### 2.1. Spontaneous LLPS Droplet Compartmentalization of Imidazolium-Copolymer in Dilution

**Figure 1:**
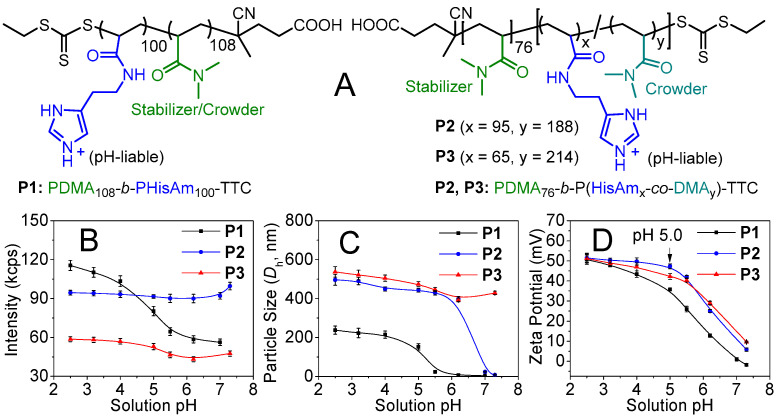
(**A**) Molecular structures of **P1** (PDMA_108_-*b*-PHisAm_100_-TTC), **P2** (PDMA_76_-*b*-P(HisAm_95_-*co*-DMA_188_)-TTC) and **P3** (PDMA_76_-*b*-P(HisAm_65_-*co*-DMA_214_)-TTC). (**B**) Light scattering intensity, (**C**) particle size (*D*_h_: number-average hydrodynamic diameter) and (**D**) zeta potential (ζ) of the **P1** (black), **P2** (blue) and **P3** (red) nanoparticles (5.0 mg/mL in water) versus solution pH. Error bars represent the standard deviation in five independent measurements.

First, we synthesized the nonionic poly(*N*,*N*-dimethylacrylamide) PDMA_108_-TTC (^1^H NMR: DP = 108; SEC: *M*_n_ = 12.1 kDa, *Đ* = 1.12; TTC: the trithiocarbonate chain-end) and PDMA_76_-TTC (^1^H NMR: DP = 76; SEC: *M*_n_ = 9.2 kDa, *Đ* = 1.17) macro-CTA through visible light initiated RAFT (photo-RAFT) polymerization [46]. Subsequently, the photo-RAFT of HisAm using PDMA_108_-TTC macro-CTA yielded poly(*N*,*N*-dimethylacrylamide)-*b*-poly(histamine acrylamide) (**P1**, PDMA_108_-*b*-PHisAm_100_-TTC; SEC: *M*_n_ = 34.7 kDa, *Đ* = 1.18), and the statistical copolymerization of HisAm and DMA using PDMA_76_-TTC macro-CTA afforded the statistical block copolymers **P2** (PDMA_76_-*b*-P(HisAm_95_-*co*-DMA_188_)-TTC; SEC: *M*_n_ = 45.3 kDa, *Đ* = 1.25) and **P3** (PDMA_76_-*b*-P(HisAm_65_-*co*-DMA_214_)-TTC; SEC: *M*_n_ = 43.6 kDa, *Đ* = 1.28), with the molecular structures shown in Figure 1A. The chain-extension experiments confirmed the high degree of the RAFT chain-end fidelity (Figures S1–S5). Unlike the **P1** PHisAm homoblock, substantial DMA units were inserted in the **P2** and **P3** PHisAm segments. This composition character was termed as “substantial DMA-dilution” for the macromolecular crowding and confinement discussed below.

Potentiometric titration results (Figure S6) indicate a decrease in the acid dissociation constant (p*K*_a_) of the imidazole group from 7.25 (monomer) to 6.26 (**P1**, Δp*K*_a_ = −1.0 pH unit), 6.50 (**P2**, −0.75 unit), and 6.42 (**P3**, −0.83 unit). Upon solution neutralization, the **P1** dispersion turns bluish (Figure S7) due to phase separation. ^1^H NMR analysis confirmed the (de)ionization and hydration of the phase-separated **P1** imidazole/imidazolium motifs (Figure S8).

Figure 1B exhibits that the light scattering intensity of the 5 mg/mL dispersion at pH 2.5–7.3 (43–116 kcps) is notably higher than that of pure water (0.66 kcps), as determined by dynamic light scattering (DLS), indicating the phase separation in charged imidazolium form. Distinct from the anti-sigmoidal intensity plot of **P1** dispersion, the intensities of the **P2**, **P3** dispersions are relatively constant due to the “substantial DMA-dilution” -induced swelling. Their number- and intensity-average hydrodynamic diameters (*D*_h_) are different (Figure S9) due to the hierarchical nanostructures discussed below. Solution neutralization leads to the decreased **P1** and **P2** particle sizes (Figure 1C). However, the *D*_h_ of **P3** particles decreases slightly due to the swelling that was caused by the “substantial DMA-dilution”. Zeta potential (ζ) analysis (Figure 1D) illustrates the charged particles with ζ > 35 mV in water at pH 2.5–5.0, above which the absolute values of ζ-potential decrease due to imidazolium deionization. The particles show excellent colloidal stability due to the steric stabilization of the hydrophilic PDMA block.

**Figure 2:**
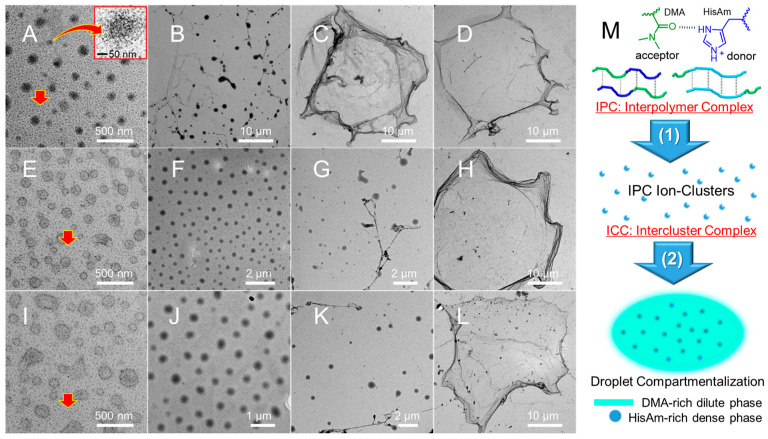
(**A**–**L**) Cryofixation TEM images (arrow: ion-cluster) of copolymer particles. (**A**–**D**) **P1** at (**A**) pH 2.5, (**B**) pH 5.0, (**C**) pH 5.5, and (**D**) pH 7.0; (**E**–**H**) **P2** at (**E**) pH 2.5, (**F**) pH 5.0, (**G**) pH 5.5, and (**H**) pH 7.0; (**I**–**L**) **P3** at (**I**) pH 2.5, (**J**) pH 5.0, (**K**) pH 5.5 and (**L**) pH 7.0. (**M**) Sketch showing hydrogen-bonded hierarchical IPC-to-ICC LLPS droplet compartmentalization, (top) blue lines denote PHisAm blocks, light blue lines denote P(HisAm-*co*-DMA) statistical blocks, green lines denote PDMA blocks, and dashed black lines denote hydrogen bonds.

The nanostructures were examined using transmission electron microscopy (TEM), using a *cryofixation technique* that primarily involved immersing the sample in liquid nitrogen and freeze-drying under high vacuum. Figure 2 displays **P1** droplets/dots **A** (pH 2.5, *inset*: dots in droplet), droplets/ribbons **B** (pH 5.0), and lamellae **C**,**D** (pH 5.5, pH 7.0). Additionally, Figure S10 exhibits numerous dots **A**. TEM statistical analysis (Figure S11) gave a number-average particle diameter (*D*_n_) of 116 ± 43 nm for droplets and 10 ± 5 nm for dots. These dots were identified as chain-collapsed ion-clusters. Given that both nonionic DMA and ionized HisAm [47] homopolymers are water-soluble, the phase separation of their copolymers can thus be ascribed to (1) *interpolymer complexation* (IPC) [40] driven by cooperative hydrogen bonding between the DMA (acceptor) and HisAm (donor) segments, resulting in complex ion-clusters; (2) *intercluster complexation* (ICC) by a delicate balance of the charge repulsion and multivalent hydrogen bonds, leading to the LLPS droplet compartmentalization into pitaya-shaped droplets (Figure 2M). Therefore, the imidazolium-block copolymer underwent phase separation via a unique *hierarchical IPC-to-ICC LLPS droplet compartmentalization* mechanism.

The dynamic properties of **P2** and **P3** droplets are enhanced by the “substantial DMA dilution”. Even given the droplet Ostwald ripening and transition to lamellae (Figure 2E–L), the coexisting light gray-colored dots and bubble-like droplets **E**, **I** (pH 2.5) indicate the notable swelling. The droplet fluidity is indicated by the bubble images reminiscent of the droplet fusion/dripping/surface wetting [48] and new formations (Figure S12). Specifically, at pH 2.5, **P2** bubbles (*D*_n_ = 95 ± 26 nm)/clusters **E** (*D*_n_ = 11 ± 6 nm, Figure S13), along with swelling **P3** bubbles (*D*_n_ = 100 ± 34 nm)/clusters (16 ± 6 nm, Figure S15) **I**, demonstrate the IPC-to-ICC LLPS droplet compartmentalization. At pH 5.0, uniform droplets **F** (*D*_n_ = 277 ± 77 nm, Figure S14) and **J** (*D*_n_ = 320 ± 73 nm, Figure S16) indicate the ICC-driven LLPS and droplet Ostwald ripening. At pH 7.0, lamellae **H**, **L** signify droplet aging caused by imidazole deionization. The aged compartments are unsuitable for the catalytic purpose due to the loss of dynamic properties. Both the “substantial DMA-dilution” and the “imidazoliumionization” facilitate the hierarchical IPC-to-ICC LLPS droplet compartmentalization, leading to the catalytic coacervates for photo-RAFT in dilution discussed below.

In summary, we achieved IPC-to-ICC LLPS droplet compartmentalization of block and statistical block imidazolium-copolymers. It is well-known that cooperative hydrogen bonding between complementary polymers results in stimulus-responsive interpolymer complexes (IPCs) [40,49,50,51]. However, to the best of our knowledge, this is the first report on hydrogen-bonded hierarchical LLPS droplet compartmentalization. The charged *dense-phase* and neutral *dilute-phase* compartments endow droplets with a high efficiency in the site-specific recruitment of oppositely charged L–AspAm monomers discussed below.

### 2.2. Site-Specific Monomer Recruitment and Localization into Droplet Compartments

**Figure 3:**
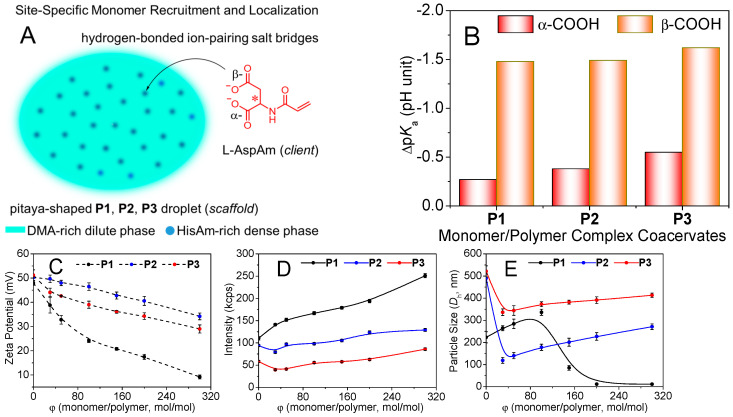
(**A**) Sketch showing the site-specific monomer recruitment into **P1**–**P3** droplet *dense-phase* compartments. (**B**) p*K*_a_ perturbations (Δp*K*_a_) of α-, β-COOH by uptake at monomer/polymer molar ratio φ = 50:1 at 30 mM (L−AspAm + HisAm units). (**C**) ζ-potential, (**D**) light scattering intensity, and (**E**) particle size versus the composition (φ) of complex coacervates at 5.0 mg/mL polymer at pH 3.2. Error bars represent the standard deviation of five independent measurements. * denotes chiral carbon.

It is well-documented that the liquid-like coacervate droplets can create an intriguing microenvironment for reactant recruitment/enrichment/localization, and destabilizing the reactants and lowering transition states to enhance reaction catalysis [14,36]. Motivated by these compelling findings, we sought to explore the reaction catalysis through the site-specific recruitment/localization of monomers into droplet *dense-phase* compartments under dilute conditions (Figure 3A). The process was driven by the COO^−^/imidazolium^+^ ion-pairing [43] biologically known as salt bridges [41,44].

We first examined the p*K*_a_ shift of ionizable groups when mixing L−AspAm monomer with the copolymer dispersions at a monomer/polymer molar ratio (φ) of 50:1 at 30 mM (L−AspAm + HisAm units). This combination caused modest decreases in the p*K*_a_ of α-COOH (L−AspAm) from 2.25 to 1.98, 1.87, and 1.70, but significant decreases in the p*K*_a_ of β-COOH from 4.13 to 2.65, 2.64, and 2.51 (Figure S17). Figure 3B illustrates the p*K*_a_ shifts indicating a notable increase in acidity. Additionally, imidazole buffer zooms (see those of precursor droplets in Figure S6) vanished due to an elevation in imidazole basicity. The concurrent enhanced COOH acidity and imidazole basicity enable dominant COO^−^•••imidazolium^+^ salt bridges for site-specific monomer recruitment and localization to oppositely charged droplet *dense-phase* compartments.

This observation well aligns with the notion that apolar environments (that is, DMA-rich dilute phase) strengthen charge–charge interactions and destabilize transition states involving charge separation. This mechanism is commonly seen in enzyme active sites [52]. Generally, protein folding induces the p*K*_a_ perturbations of ionizable motifs [53,54,55]. For example, a salt bridge forms between Asp-70 and His-31 during T4 lysozyme folding [53], leading to notable p*K*_a_ shifts of COOH from 3.5–4.0 to 0.5 and of imidazole from 6.8 to 9.1. Biologically, p*K*_a_ perturbations play key roles in enzyme solubility and stability and functions such as kinetic properties [54]. The following study therefore focused on salt-bridged monomer recruitment at pH 3.2. Theoretically, the monomer/polymer complex coacervates contain 100% imidazolium and >90% COO^−^ motifs (>98% α-, ~80% β-COOH ionized, Figure S18), facilitating the salt-bridging molecular recognition.

To elucidate the composition (φ) impacts on the complex coacervation, we prepared a range of the mixtures at 5.0 mg/mL polymer at pH 3.2, with φ ranging from 30 to 300. The dispersions are transparent with perfect long-term storage stability. Figure 3C shows a ζ-shift of **P1** parent droplets from ζ = 48 mV close to isoelectric point (9.2 mV) at φ = 300, but **P2** and **P3** droplets show a slight ζ-shift due to the “substantial DMA-dilution”. The **P1** dispersion exhibits a dramatic increase in light scattering intensity, but **P2**, **P3** dispersions show a mild increase in intensity (Figure 3D). Figure 3E exhibits an anti-sigmoidal variation of **P1** particle size, which is associated with the charge-balanced morphological transition to the solid-like lamellae, as discussed below. Nevertheless, the sizes of the “substantial DMA-dilution” -featured **P2** and **P3** particles decrease sharply at φ = 0–30 but increase mildly at 30–300 due to the droplet shrinkage and Ostwald ripening.

**Figure 4:**
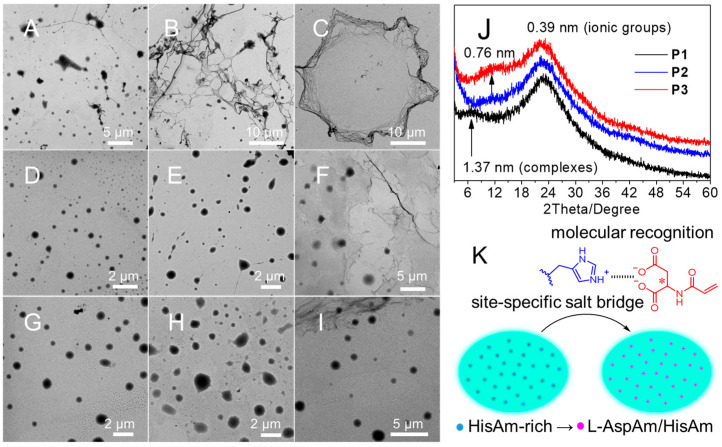
(**A**–**I**) Cryofixation TEM images of **P1** monomer complex coacervates at φ = 30 (**A**), 50 (**B**), and 100 (**C**); **P2** at φ = 30 (**D**), 50 (**E**), and 100 (**F**); **P3** at φ = 30 (**G**), 50 (**H**), and 100 (**I**), at pH 3.2. (**J**) XRD patterns of the **P1**, **P2**, **P3** monomer coacervates at φ = 50 at pH 3.2. (**K**) Sketch showing site-specific monomer recruitment/localization to the *dense-phase* compartments within apolar DMA-rich liquid-like *dilute-phase* environments via salt-bridging molecular recognition. * denotes chiral carbon.

The droplets were freeze-dried and examined using TEM and X-ray diffraction (XRD) analyses. Figure 4 displays Ostwald ripened **P1** droplets **A** (φ = 30), and lamellae **B** (50)/**C** (100), suggesting a strong solidification trend, which halt the diffusion-limited reaction due to reduced fluidity. However, the “substantial DMA-dilution” leads to **P2** droplets **D**, **E** (30–50), droplets/lamellae **F** (100), and **P3** droplets **G**, **H**, **I** (30–100). Figure 4J reveals the amorphous structures with features at *d* = 0.39 nm corresponding to COO^−^/Na^+^ and imidazolium^+^/Cl^−^ salts [56,57]. Moreover, the *d*-spacing of ion-pairing complexes decreases from 1.37 (**P1**) to 0.76 nm (**P2**, **P3**), suggesting the “substantial DMA-dilution” -induced size reduction. All of these results demonstrate that the salt-bridging molecular recognition facilitates site-specific monomer recruitment/localization to the oppositely charged *dense-phase* compartments maintaining apolar DMA-rich *dilute-phase* crowding environments, as depicted in Figure 4K.

### 2.3. Kinetic Properties of Catalytic Coacervates for Photo-RAFT in Dilution

As mentioned above, we achieved the site-specific monomer recruitment/localization into the scaffold droplet *dense-phase* compartments, resulting in droplets at low φ in water at pH 3.2. These intriguing results inspired us to explore coacervate reaction catalysis [14]. Accordingly, we studied the kinetic properties of these active coacervates, under aqueous photo-RAFT conditions, in <2% *w*/*w* monomer dilution at 25 °C.

**Figure 5:**
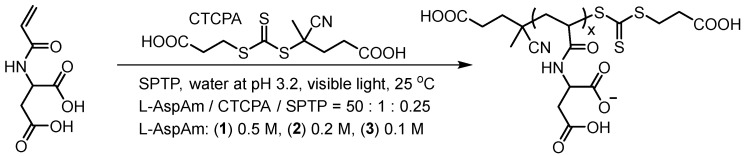

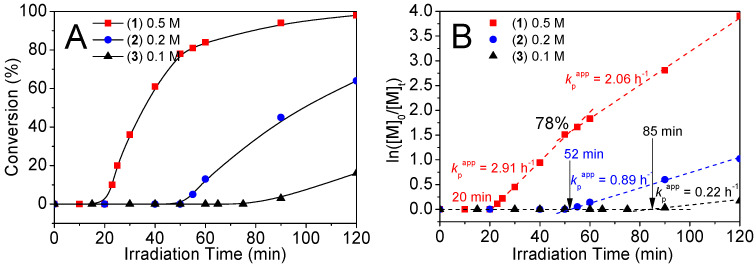
Kinetic plots of aqueous photo-RAFT polymerization of L−AspAm using a CTCPA small chain transfer agent (control experiment without copolymer droplets). (**A**) Conversion and (**B**) ln([M]_0_/[M]_t_) evolution with irradiation time.

As a control experiment (Figure 5, top scheme), we studied the dilution effects on the photo-RAFT polymerization varying (**1**) 0.5, (**2**) 0.2, and (**3**) 0.1 M L–AspAm concentration. Conversion was determined using ^1^H NMR (Figure S19), and the growing polymers were studied by aqueous size exclusion chromatography (SEC, Figure S20). Figure 5A shows remarkable concentration impacts. Conversion decreased from 98% (0.5 M L–AspAm) to 64% (0.2 M), 16% (0.1 M) after the 2-h light irradiation. Figure 5B shows the distinctive dilution-prolonged induction time. Linear semi-logarithmic plots suggesting the constant concentration of growing-chain radicals. The clear shift of SEC trace and low dispersity (*Đ* ≤ 1.17) of the growing polymers (Figure S20) suggest the well-controlled photo-RAFT polymerization. The results demonstrate that the dilution not only increase the induction time but also decrease the chain propagation rate of this well-controlled polymerization, which is unfavorable for the dilution-compulsory practical applications.

**Figure 6:**
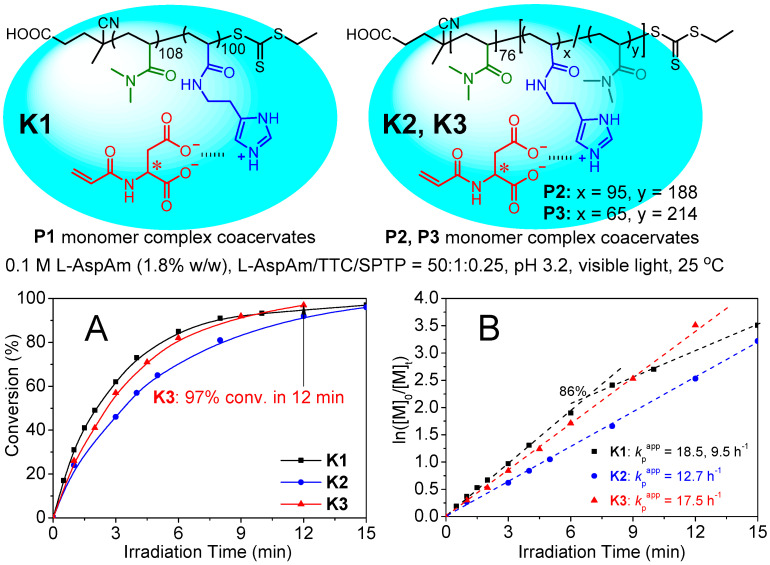
Kinetics of the polymerization of the **P1** (**K1**, black), **P2** (**K2**, blue), and **P3** (**K3**, red) monomer complex coacervates (**top scheme**). (**A**) Conversion, (**B**) ln([M]_0_/[M]_t_) evolution with irradiation time. * denotes chiral carbon.

Thereafter, we sought to explore the catalytic coacervates to speed up aqueous photo-RAFT in dilution. As shown in top scheme of Figure 6, the kinetic properties were studied through the following steps: *(1) Monomer Recruitment* involved mixing L−AspAm monomer with copolymer dispersions at φ = 50 (target DP) at 0.10 M monomer (1.8% *w*/*w*) at pH 3.2, and stirring overnight. *(2) Heterogeneous Polymerization* involved transferring the stock solution (1.0 mL) to a reaction flask, adding the SPTP photoinitiator (TTC/SPTP = 1:0.25), deoxygenating with argon gas in darkness, and finally exposing to visible light at 25 °C. Conversion was determined using ^1^H NMR (Figure S21). Reaction kinetics of the **P1**, **P2**, and **P3** monomer complex coacervates are denoted as **K1**, **K2**, and **K3** respectively.

Figure 6A shows immediate polymerization without the induction period observed in the discussed control experiment. Moreover, a rapid chain propagation is evidenced by a 97% conv. in 12 min. Figure 6B shows a rapid–slow **K1** kinetic plot with apparent chain propagation rate constant (*k*_p_^app^) of 18.6, 9.5 h^−1^, dramatically higher than 0.22 h^−1^ of the 0.1 M counterpart (Figure 5B). The onset of the reaction deceleration occurred at 86% conv., exceeding 78% conversion in the 0.5 M counterpart (Figure 5B). Generally, in traditional RAFT polymerization, dilution slows down reaction, and low target DPs lead to notable reaction retardation by the intermediate reversible addition-fragmentation reactions [58]. Nevertheless, in this reaction in 1.8% *w*/*w* monomer dilution at low target DP of 50, the monomer was consumed promptly and swiftly. This reaction catalysis can thus be ascribed to site-specific monomer recruitment/localization to the droplet *dense-phase* compartments.

Figure 6B shows **K2**, **K3** linear semi-logarithmic kinetic plots, where **K3** reached 97% conversion in 12 min (see Figure 6A). The **K3** (*k*_p_^app^ = 17.5 h^−1^), faster than the **K2** (12.7 h^−1^), highlights the critical role of the liquid-like dynamic properties for the reaction catalysis. All of these results demonstrate that the reaction catalysis is driven by site-specific monomer recruitment/localization into the droplet *dense-phase* compartments allowing liquid *dilute-phase* crowding environments to eliminate the kinetically trapped slow reaction.

**Figure 7:**
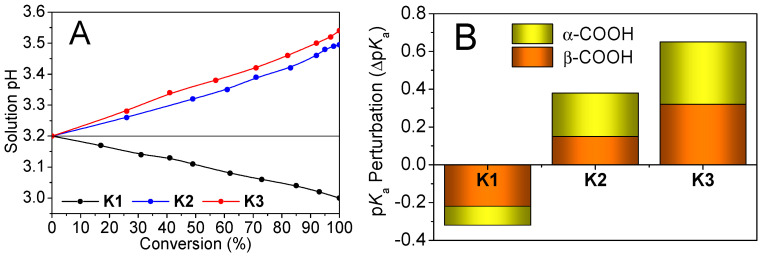
(**A**) Solution pH-shift during **K1**–**K3** polymerization. (**B**) p*K*_a_ perturbation (Δp*K*_a_) of α- and β-COOH groups (L−AspAm) upon **K1**–**K3** polymerization up to 100% conversions.

At over 41% conversions, the **K1** dispersion became bluish (Figure S22) suggesting the particle size increase. In contrast, the **K2** and **K3** dispersions remained transparent due to the “substantial DMA-dilution”. Furthermore, reaction resulted in **K1** solution acidification but **K2**, **K3** solution neutralization (Figure 7A). The **K1** final product displays p*K*_a_ of 1.76 (α-), 2.56 (β-) for COOH motifs, notably lower than the **Asp100** homopolymer counterpart, with substantial p*K*_a_ decrease (Δp*K*_a_: α- > 2 pH units, β- > 4 units, Figure S23). Significant p*K*_a_ perturbations are also observed in the **K2** and **K3** final products, suggesting substantial COO^−^•••imidazolium^+^ salt bridges in the final products. Furthermore, compared to the monomer/polymer coacervates (see Figure S17), the **K1** final COOH motifs show negative p*K*_a_ shift, whilst those in the **K2**, **K3** products show a positive p*K*_a_ shift (Figure 7B). The final imidazole buffer zooms re-emerged, with the p*K*_a_ = 7.76 (**K2** final) and 7.53 (**K3** final) lower than that (8.38) of the **K1** final due to the “substantial DMA-dilution”. The opposing trends of proton feedbacks (release vs. recruitment) led to **K1** reaction solution acidification, but **K2** and **K3** reaction solution neutralization.

**Figure 8:**
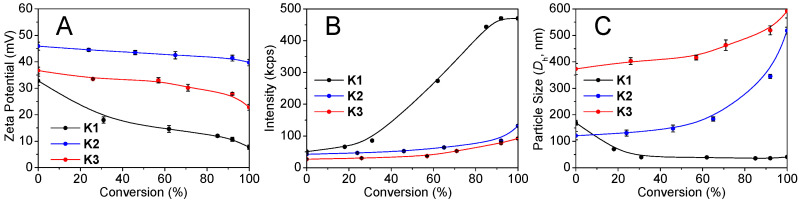
(**A**) Zeta potential, (**B**) light scattering intensity and (**C**) particle size of **K1**–**K3** coacervates versus conversions. Error bars represent the standard deviation of five independent measurements.

Figure 8A shows a decrease in ζ-potential for **K1** droplets at 0–30% conversion, and then a gradual decrease due to the lamella formation discussed below. However, **K2** and **K3** droplets show a mild ζ-decrease owing to “substantial DMA-dilution” for the *dilute-phase* swelling and *dense-phase* sequestration. Figure 8B,C shows the intensity increase and size decrease of **K1** coacervates caused by the droplets-to-lamellae transition discussed below. The low intensity but slight size increase of **K2** and **K3** droplets can be attributed to the considerable swelling caused by the “substantial DMA-dilution”.

**Figure 9:**
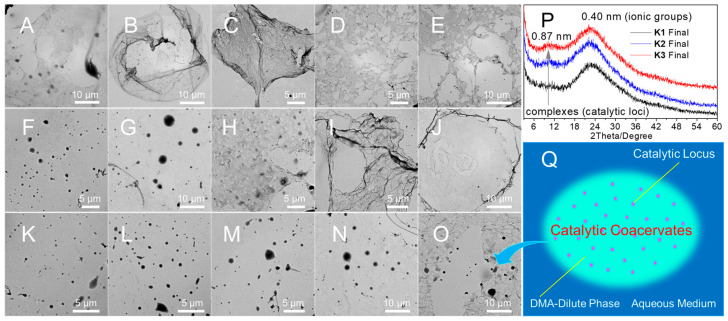
Cryofixation TEM images of (**A**–**E**) **K1** coacervates at (**A**) 17%, (**B**) 31%, (**C**) 49%, (**D**) 85%, and (**E**) 100% conversions; (**F**–**J**) **K2** at (**F**) 24%, (**G**) 46%, (**H**) 65%, (**I**) 92%, and (**J**) 100% conversions; (**K**–**O**) **K3** at (**K**) 26%, (**L**) 57%, (**M**) 71%, (**N**) 92%, and (**O**) 100% conversions. (**P**) XRD patterns of **K1**–**K3** final products. (**Q**) Sketch showing the internal structures of catalytic coacervates.

Finally, we examined reaction coacervates using cryofixation TEM and XRD. Figure 9 indicates the “substantial DMA-dilution” liquefaction, as illustrated by the transitions from **K1** droplets/lamellae **A** (17% conv.) to lamellae **B**–**E** (31–100%), from **K2** droplets **G** (46%) to droplets/lamellae **H** (65%) to lamellae **I**, **J** (92–100%), and from **K3** droplets **K**–**M** (26–71%) to droplets/lamellae **N**, **O** (92–100%). The kinetically trapped reaction observed in **K1** was eliminated by liquefying the **K2** and **K3** coacervates. Additionally, Figure 9P reveals 0.87-nm *d*-spacing in **K2** and **K3** final products indicating sustainable *dense-phase* catalytic reaction sites. Another factor contributing to the reaction catalysis is the macromolecular crowding and confinement [37,39] by the DMA-rich *dilute-phase* crowding surroundings. The excluded volume effect [37,39] promotes growing chain compaction, facilitating chain propagation by reducing volume compared to monomers [14]. Consequently, the “substantial DMA-dilution” results in the sustainable catalytic sites in the apolar DMA-rich liquid-like *dilute-phase* crowding environments, as depicted in Figure 9Q.

In short, we developed the catalytic coacervates for photo-RAFT in dilution via site-specific monomer recruitment and localization, with the aid of macromolecular crowding and confinement. The “substantial DMA-dilution” ensured the sustainable catalytic sites within liquid-like apolar *dilute-phase* crowding environments, resulting in 97% conversion within 12 min. Sumerlin’s group [59] introduced an ultrafast UV-photoiniferter PISA; however, when targeting a DP of 50, the reaction slowed due to the lack of monomer compartmentalization in the micellar core [59]. Boyer and coworkers [60] achieved an ultrafast photoiniferter PISA under visible light, but at 50% *v*/*v* solids content. Shi [61] succeeded in an ultrafast ring-opening metathesis polymerization by means of monomer emulsification. An’s group [62,63,64] has demonstrated the remarkably high efficiencies of enzyme-catalyzed RAFT polymerization. Nevertheless, catalytic photo-RAFT through enzyme-like droplet compartmentalization for site-specific monomer recruitment/localization in dilution has hitherto been unreported. These findings underscore a key role of macromolecular crowding and confinement in the LLPS droplet compartmentalization and site-specific monomer recruitment/localization, and provide a practical guideline for rational design of catalytic coacervates toward lifelike reaction functionalities.

## 3. Conclusions

In summary, we developed catalytic coacervates for aqueous photo-RAFT in dilution, focusing on the site-specific monomer recruitment/localization through macromolecular crowding and confinement. Our results revealed that both the block and statistical block copolymers underwent hydrogen-bonded interpolymer complexes, resulting in complex ion-clusters. Intercluster complexation led to LLPS and droplet compartmentalization into charged *dense-phase* and neutral *dilute-phase* compartments. These droplets exhibited the capacity of site-specific monomer recruitment and localization via salt-bridging molecular recognition. The “substantial DMA-dilution” offered liquid-like apolar *dilute-phase* crowding surroundings for the sustainable *dense-phase* catalytic reaction sites, enabling the reaction catalysis to 97% conversion in 12 min. Notably, the strategies used for catalytic coacervates mirror those seen in enzyme active sites, such as creating apolar microenvironments, perturbing p*K*_a_ of ionizable groups, and involving proton feedback mechanisms. Given that these active coacervates originated from an amino acid monomer, we propose that the active coacervates could represent rudimentary precursors of synthetic enzymes. These findings underscored the key role of macromolecular crowding and confinement in the LLPS droplet compartmentalization and site-specific monomer recruitment/localization that are essential for enzyme reaction catalysis, and provided practical guidelines for creating the catalytic coacervates toward lifelike reaction functionalities.

## Data Availability

The raw data supporting the conclusions of this article will be made available by the authors on request.

## Supplementary Materials

The following supporting information can be downloaded at: https://www.mdpi.com/article/10.3390/polym18010106/s1, Experimental Section [46,47,65,66,67]; Figures S1–S5: ^1^H NMR spectra, SEC traces; Figure S6: titration plots; Figure S7: digital photos; Figure S8: ^1^H NMR spectra; Figure S9: number-, intensity-average size distribution; Figure S10: TEM images; Figure S11: TEM statistical analysis data; Figure S12: TEM images; Figures S13–S16: TEM statistical analysis data; Figure S17: titration plots; Figure S18: degrees of ionization; Figure S19: ^1^H NMR spectra; Figure S20: SEC traces; Figure S21: ^1^H NMR spectra; Figure S22: digital photos; Figure S23: titration plots.

## References

[1] Shin Y., Brangwynne C.P. (2017). Liquid phase condensation in cell physiology and disease. Science.

[2] Dignon G.L., Best R.B., Mittal J. (2020). Biomolecular phase separation: From molecular driving forces to macroscopic properties. Annu. Rev. Phys. Chem..

[3] Alberti S., Gladfelter A., Mittag T. (2019). Considerations and challenges in studying liquid-liquid phase separation and biomolecular condensates. Cell.

[4] Galvanetto N., Ivanović M.T., Chowdhury A., Sottini A., Nüesch M.F., Nettels D., Best R.B., Schuler B. (2023). Extreme dynamics in a biomolecular condensate. Nature.

[5] Hyman A.A., Weber C.A., Juelicher F. (2014). Liquid-liquid phase separation in biology. Annu. Rev. Cell Dev. Biol..

[6] Banani S.F., Lee H.O., Hyman A.A., Rosen M.K. (2017). Biomolecular condensates: Organizers of cellular biochemistry. Nat. Rev. Mol. Cell Biol..

[7] Hanopolskyi A.I., Mikhnevich T.A., Paikar A., Nutkovich B., Pinkas I., Dadosh T., Smith B.S., Orekhov N., Skorb E.V., Semenov S.N. (2023). Interplay between autocatalysis and liquid-liquid phase separation produces hierarchical microcompartments. Chem.

[8] The Nobel Prize in Chemistry 2009 (Information for the Public)—The Key to Life at the Atomic Level. https://www.nobelprize.org/uploads/2018/06/popular-chemistryprize2009.pdf.

[9] Blocher W.C., Perry S.L. (2017). Complex coacervate-based materials for biomedicine. WIREs Nanomed. Nanobiotechnol..

[10] Qin J., Priftis D., Farina R., Perry S.L., Leon L., Whitmer J., Hoffmann K., Tirrell M., de Pablo J.J. (2014). Interfacial tension of polyelectrolyte complex coacervate phases. ACS Macro Lett..

[11] Koide A., Kishimura A., Osada K., Jang W.D., Yamasaki Y., Kataoka K. (2006). Semipermeable polymer vesicle (PICsome) self-assembled in aqueous medium from a pair of oppositely charged block copolymers: Physiologically stable micro-/nanocontainers of water-soluble macromolecules. J. Am. Chem. Soc..

[12] Anraku Y., Kishimura A., Yamasaki Y., Kataoka K. (2013). Living unimodal growth of polyion complex vesicles via two-dimensional supramolecular polymerization. J. Am. Chem. Soc..

[13] Aumiller W.M., Keating C.D. (2016). Phosphorylation-mediated RNA/peptide complex coacervation as a model for intracellular liquid organelles. Nat. Chem..

[14] Smokers I.B.A., Visser B.S., Slootbeek A.D., Huck W.T.S., Spruijt E. (2024). How droplets can accelerate reactions-coacervate protocells as catalytic microcompartments. Acc. Chem. Res..

[15] Abbas M., Lipinski W.P., Nakashima K.K., Huck W.T.S., Spruijt E. (2021). A short peptide synthon for liquid-liquid phase separation. Nat. Chem..

[16] Mukhopadhyay S. (2021). Catalytic coacervate crucibles. Nat. Chem..

[17] Fan Y., Tang S.C., Thomas E.L., Olsen B.D. (2014). Responsive block copolymer photonics triggered by protein-polyelectrolyte coacervation. ACS Nano.

[18] Schoonen L., van Hest J.C. (2016). Compartmentalization approaches in soft matter science: From nanoreactor development to organelle mimics. Adv. Mater..

[19] Donau C., Spath F., Sosson M., Kriebisch B.A.K., Schnitter F., Tena-Solsona M., Kang H.S., Salibi E., Sattler M., Mutschler H. (2020). Active coacervate droplets as a model for membraneless organelles and protocells. Nat. Commun..

[20] Wei Y., Cheng G., Ho H.P., Ho Y.P., Yong K.T. (2020). Thermodynamic perspectives on liquid-liquid droplet reactors for biochemical applications. Chem. Soc. Rev..

[21] An Z., Shi Q., Tang W., Tsung C.-K., Hawker C.J., Stucky G.D. (2007). Facile RAFT precipitation polymerization for the microwave-assisted synthesis of well-defined, double hydrophilic block copolymers and nanostructured hydrogels. J. Am. Chem. Soc..

[22] Wan W.-M., Hong C.-Y., Pan C.-Y. (2009). One-pot synthesis of nanomaterials via RAFT polymerization induced self-assembly and morphology transition. Chem. Commun..

[23] Blanazs A., Madsen J., Battaglia G., Ryan A.J., Armes S.P. (2011). Mechanistic insights for block copolymer morphologies: How do worms form vesicles?. J. Am. Chem. Soc..

[24] Jiang Y., Xu N., Han J., Yu Q., Guo L., Gao P., Lu X., Cai Y. (2015). The direct synthesis of interface-decorated reactive block copolymer nanoparticles via polymerisation-induced self-assembly. Polym. Chem..

[25] Tan J., Sun H., Yu M., Sumerlin B.S., Zhang L. (2015). Photo-PISA: Shedding light on polymerization-induced self-assembly. ACS Macro Lett..

[26] Yeow J., Xu J., Boyer C. (2015). Polymerization-induced self-assembly using visible light mediated photoinduced electron transfer-reversible addition-fragmentation chain transfer polymerization. ACS Macro Lett..

[27] Penfold N.J.W., Yeow J., Boyer C., Armes S.P. (2019). Emerging trends in polymerization-induced self-assembly. ACS Macro Lett..

[28] D’Agosto F., Rieger J., Lansalot M. (2020). RAFT-mediated polymerization-induced self-assembly. Angew. Chem. Int. Ed..

[29] Zhang B., Lv X., An Z. (2017). Modular monomers with tunable solubility: Synthesis of highly incompatible block copolymer nano-objects via RAFT aqueous dispersion polymerization. ACS Macro Lett..

[30] Ding Y., Cai M., Cui Z., Huang L., Wang L., Lu X., Cai Y. (2018). Synthesis of low-dimensional polyion complex nanomaterials via polymerization-induced electrostatic self-assembly. Angew. Chem. Int. Ed..

[31] Bos I., Terenzi C., Sprakel J. (2020). Chemical feedback in templated reaction-assembly networks. Macromolecules.

[32] Li C., Magana J.R., Sobotta F., Wang J., Stuart M.A.C., Van Ravensteijn B.G.P., Voets I.K. (2022). Switchable electrostatically templated polymerization. Angew. Chem. Int. Ed..

[33] Sobotta F.H., van Ravensteijn B.G.P., Voets I.K. (2025). Sequence-controlled neutral-ionic multiblock-like copolymers through switchable PIESA in a one-pot approach. ACS Macro Lett..

[34] Ding Y., Zhao Q.Q., Wang L., Huang L.L., Liu O.Z., Lu X.H., Cai Y.L. (2019). Polymerization-induced self-assembly promoted by liquid-liquid phase separation. ACS Macro Lett..

[35] Zhang R., Fan X., Xu X., Niu W., Cai Y. (2025). Self-adaptable activation and inactivation of RAFT polymerization: A liquid-liquid phase separation scaffold-client platform. Eur. Polym. J..

[36] Howlett M.G., Fletcher S.P. (2023). From autocatalysis to survival of the fittest in self-reproducing lipid systems. Nat. Rev. Chem..

[37] Zhou H.X., Rivas G.N., Minton A.P. (2008). Macromolecular crowding and confinement: Biochemical, biophysical, and potential physiological consequences. Annu. Rev. Biophys..

[38] Guin D., Gruebele M. (2019). Weak chemical interactions that drive protein evolution: Crowding, sticking, and quinary structure in folding and function. Chem. Rev..

[39] Subramanya A.R., Boyd-Shiwarski C.R. (2024). Molecular crowding: Physiologic sensing and control. Annu. Rev. Physiol..

[40] Schmidt R.F., Lutzki J., Dalgliesh R., Prévost S., Gradzielski M. (2025). pH-Responsive rheology and structure of poly(ethylene oxide)–poly(methacrylic acid) interpolymer complexes. Macromolecules.

[41] Pylaeva S., Brehm M., Sebastiani D. (2018). Salt bridge in aqueous solution: Strong structural motifs but weak enthalpic effect. Sci. Rep..

[42] Bosshard H.R., Marti D.N., Jelesarov I. (2004). Protein stabilization by salt bridges: Concepts, experimental approaches and clarification of some misunderstandings. J. Mol. Recognit..

[43] Marcus Y., Hefter G. (2006). Ion pairing. Chem. Rev..

[44] Donald J.E., Kulp D.W., DeGrado W.F. (2011). Salt bridges: Geometrically specific, designable interactions. Proteins.

[45] Buddingh B.C., van Hest J.C.M. (2017). Artificial cells: Syntheticcompartments with life-like functionality and adaptivity. Acc. Chem. Res..

[46] Shi Y., Liu G., Gao H., Lu L., Cai Y. (2009). Effect of mild visible light on rapid aqueous RAFT polymerization of water-soluble acrylic monomers at ambient temperature: Initiation and activation. Macromolecules.

[47] Cao L., Zhao Q., Liu Q., Ma L., Li C., Wang X., Cai Y. (2020). Electrostatic manipulation of triblock terpolymer nanofilm compartmentalization during aqueous photoinitiated polymerization-induced self-assembly. Macromolecules.

[48] Dogra P., Joshi A., Majumdar A., Mukhopadhyay S. (2019). Intermolecular charge-transfer modulates liquid-liquid phase separation and liquid-to-solid maturation of an intrinsically disordered pH-responsive domain. J. Am. Chem. Soc..

[49] Tsuchida E., Abe K. (1982). Interactions between macromolecules in solution and intermacromolecular complexes. Adv. Polym. Sci..

[50] Rattanawongwiboon T., Ghaffarlou M., Sütekin S.D., Pasanphan W., Güven O. (2018). Preparation of multifunctional poly(acrylic acid)-poly(ethylene oxide) nanogels from their interpolymer complexes by radiation-induced intramolecular crosslinking. Colloid. Polym. Sci..

[51] Nurkeeva Z.S., Mun G.A., Khutoryanskiy V.V., Bitekenova A.B., Dubolazov A.V., Esirkegenova S.Z. (2003). pH effects in the formation of interpolymer complexes between poly(N-vinylpyrrolidone) and poly(acrylic acid) in aqueous solutions. Euro. Phys. J. E.

[52] Nott T.J., Petsalaki E., Farber P., Jervis D., Fussner E., Plochowietz A., Craggs T.D., Bazett-Jones D.P., Pawson T., Forman-Kay J.D. (2015). Phase transition of a disordered nuage protein generates environmentally responsive membraneless organelles. Mol. Cell.

[53] Anderson D.E., Becktel W.J., Dahlquist F.W. (1990). pH-induced denaturation of proteins–A single salt bridge constitutes 3-5 kcal/mol to the free-energy of folding of T4-lysozyme. Biochemistry.

[54] Pace C.N., Grimsley G.R., Scholtz J.M. (2009). Protein ionizable groups: pK values and their contribution to protein stability and solubility. J. Biol. Chem..

[55] Palmadottir T., Malmendal A., Leiding T., Lund M., Linse S. (2021). Charge regulation during amyloid formation of alpha-synuclein. J. Am. Chem. Soc..

[56] Abou Hamad N., Akintola J., Schlenoff J.B. (2025). Quantifying hydrophilicity in polyelectrolytes and polyzwitterions. Macromolecules.

[57] Ramírez Marrero I.A., Kaiser N., von Vacano B., Konradi R., Crosby A.J., Perry S.L. (2025). Brittle-to-ductile transitions of polyelectrolyte complexes: Humidity, temperature, and salt. Macromolecules.

[58] Moad G., Rizzardo E., Thang S.H. (2008). Toward living radical polymerization. Acc. Chem. Res..

[59] Bowman J.I., Eades C.B., Vratsanos M.A., Gianneschi N.C., Sumerlin B.S. (2023). Ultrafast xanthate-mediated photoiniferter polymerization-induced self-assembly (PISA). Angew. Chem. Int. Ed..

[60] Wu Z.L., He Z.Y., Zhou Y.C., Kou T.T., Gong K.L., Nan F.C., Bezuneh T.T., Han S.G., Boyer C., Yu W.W. (2025). Design of an ultrafast and controlled visible light-mediated photoiniferter RAFT polymerization for polymerization-induced self-assembly (PISA). Angew. Chem. Int. Ed..

[61] Hou W., Yin X., Chen Y., Du J., Chen Y., Shi Y. (2023). Ultrafast monomer emulsified aqueous ring-opening metathesis polymerization for the synthesis of water-soluble polynorbornenes with precise structure. ACS Macro Lett..

[62] Li R., Kong W., An Z. (2022). Enzyme catalysis for reversible deactivation radical polymerization. Angew. Chem. Int. Ed..

[63] Zhao Y., Zhang S.D., An Z.S. (2025). Amplified cascade catalysis for RAFT polymerization. Angew. Chem. Int. Ed..

[64] Li R.Y., Zhang S.D., Tang X.H., Qiao G.G., Armes S.P., An Z.S. (2025). Near-infrared photoenzymatic catalysis at ppb levels enables ultrahigh-molecular-weight polymers. J. Am. Chem. Soc..

[65] Thang S.H., Chong Y.K., Mayadunne R.T.A., Moad G., Rizzardo E. (1999). A novel synthesis of functional dithioesters, Dithiocarbamates, xanthates and trithiocarbonates. Tetrahedron Lett..

[66] Luo C., Wang X., Liu Y., Cai J., Lu X., Cai Y. (2023). Like-charge PISA: Polymerization-induced like-charge electrostatic self-assembly. ACS Macro Lett..

[67] Bronstert B., Henne A., Hesse A., Jacobi M., Wallbillich G. (1988). Acylphosphine Compounds and their Use as Photoinitiators. U.S. Patent.

