# Supplementary material for: Site-Specific Recruitment, Localization of Ionized Monomer to Macromolecular Crowded Droplet Compartments Can Lead to Catalytic Coacervates for Photo-RAFT in Dilution
Source: Polymers (Basel). 2025 Dec 30;18(1):106. doi: 10.3390/polym18010106 (PMC12787518; doi:10.3390/polym18010106)
Supplement: Supplementary file 1 [file polymers-18-00106-s001.zip › polymers-4062208-supplementary.pdf]

Site-Specific Recruitment, Localization of Ionized Monomer to Macromolecular Crowded Droplet Compartments Can Lead to Catalytic Coacervates for Photo-RAFT in Dilution

*Wenjing Niu, Xiyu Wang, Ran Zhang and Yuanli Cai**

State-Local Joint Engineering Laboratory for Novel Functional Polymer Materials, Jiangsu Key Laboratory of Advanced Functional Polymer Materials, Suzhou Key Laboratory of Macromolecular Design and Precision Synthesis, College of Chemistry, Chemical Engineering and Materials Science, Soochow University, Suzhou 215123, China

Supplementary Materials

Experimental Section

Materials. 4-Cyano-4-ethylsulfanylthiocarbonylsulfanylpentanoic acid[1] (CEP) chain transfer agent was synthesized using literature procedures. The L-AspAm monomer and **Asp100** homo-polymer[2] (^{1}H NMR: DP = 100; SEC: M_n = 18.1 kDa, $Đ$ = 1.24), HisAm [3] monomer and sodium phenyl-2,4,6-trimethylbenzoylphosphinate (SPTP)[4] initiator were synthesized previously. DMA monomer was purchased from Beijing Warwick Chem. Co. 4-2-Carboxyethylthiocarbonothioyl-thio-4-cyanopentanoic acid (CTCPA) chain transfer agent was purchased from Sigma-Aldrich. Dimethylsulfoxide-d$_6$ (DMSO-d$_6$, 99.9% D), deuterium oxide (D_2O, 99.8% D), deuterium chloride (20% in D_2O, 99.5% D), and sodium deuteroxide (40% in D_2O, 99.9% D) were obtained from J&K. 2,2-Dimethyl-2-silapentane-5-sulfonate sodium salt (DSS) was obtained from TCI. Diphenyl(2,4,6-trimethylbenzoyl)-phosphine oxide (TPO) initiator and other reagents were obtained from Aladdin. DMA monomer was purified by passing it through a basic aluminum oxide column to eliminate inhibitor. All other reagents were used as received. Milli-Q water (R > 18.2 MΩ/cm) was obtained using a Direct-Q5 UV Millipore system, and was employed in the synthesis and characterization processes.

Synthesis of PDMA-TTC Macro-CTA. This macro-CTA was synthesized by photo-RAFT[5] using following procedures. Typically, CEP chain transfer agent (0.132 g, 0.50 mmol) and DMA monomer (9.913 g, 0.10 mol) were dissolved in methanol (10.091 g) in a 50-mL reaction flask. Subsequently, TPO initiator (61.0 mg, 0.18 mmol) was added to the flask in darkness. The flask was immersed in a water bath at 25 °C. After purged with argon gas for 45 min, the flask was exposed to visible light for 45 min. The reaction was quenched by exposure to air and the addition of hydroquinone inhibitor. ^{1}H NMR: 53% conv. The polymer was precipitated from cold ethyl ether, and dried under vacuum. Weight: 4.521 g, Yield: 85%. ^{1}H NMR: DP = 108; SEC: M_n = 12.1

kDa, $Đ$ = 1.12; denoted as $PDMA_{108}$-TTC (TTC: trithiocarbonate chain-ends, number: DP). The $PDMA_{76}$-TTC macro-CTA was synthesized under the same conditions but at DMA/CEP/SPTP = 100:1:0.35 under visible light for 200 min. ^{1}H NMR: 72% conv. Weight: 6.212 g, Yield: 85%. ^{1}H NMR: DP = 76; SEC: M_n = 9.2 kDa, $Đ$ = 1.17. The yield was calculated according to Eq. S1, in which $W_{polymer}$, W_{CEP} and W_{DMA} denote the weights of the purified polymer, CEP chain transfer agent and DMA monomer.

$$\text{Yield} = \frac{W_{polymer}}{(W_{CEP}+W_{DMA})\times conversion} \times 100\% \qquad \text{(S1)}$$

Synthesis of Imidazolium-Block Copolymer. $PDMA_{108}$-TTC macro-CTA (0.877 g, 81 μmol) and HisAm monomer (1.337 g, 8.1 mmol) were dissolved in methanol/water (3/7 w/w, 3.320 g) in a 10-mL reaction flask. The solution was adjusted to pH 2.5. SPTP initiator (8.8 mg, 0.03 mmol) was added to the flask in darkness. The flask was immersed in a water bath at 25 °C. The solution was purged with argon for 45 min. The flask was expose to visible light for 20 min. The reaction was quenched by exposure to air and addition of hydroquinone inhibitor. ^{1}H NMR: 100% conv. The solution was dialyzed using a dialysis tube (MWCO = 1.0 kDa), lyophilized to afford the product. Weight: 2.051 g, Yield: 93%. ^{1}H NMR: $PDMA_{108}$-*b*-$PHisAm_{100}$-TTC; SEC: M_n = 34.7 kDa, $Đ$ = 1.18. This block copolymer was denoted as **P1**. The yield was calculated based on Eq. S2, where $W_{polymer}$, $W_{macro\text{-}CTA}$ and W_{HisAm} denote the weights of the purified polymer, $PDMA_{108}$-TTC macro-CTA and HisAm monomer.

$$\text{Yield} = \frac{W_{polymer}}{(W_{macro-CTA}+W_{HisAm})\times conversion} \times 100\% \qquad \text{(S2)}$$

Synthesis of Statistical Block Imidazolium-Copolymers with "Substantial DMA-Dilution". Typically, HisAm (0.759 g, 4.6 mmol) and DMA (1.498 g, 15.1 mmol) monomers, and $PDMA_{76}$-TTC macro-CTA (0.515 g, 66 μmol) were dissolved in methanol/water (3/7 w/w, 2.772 g) in a 10-mL flask. The solution was adjusted to pH 2.5. Subsequently, SPTP initiator (5.1 mg, 16 μmol)

was added to the flask in darkness. The flask was immersed in a water bath at 25 °C. After purged with argon for 45 min, the flask was exposed to visible light for 6 min. The reaction was quenched by exposure to air and addition of hydroquinone inhibitor. ^{1}H NMR: 93% DMA and 93% HisAm conv. The solution was dialyzed using a dialysis tube and freeze-dried to afford the product. Weight: 2.410 g, Yield: 93%. ^{1}H NMR: PDMA$_{76}$-*b*-P(HisAm$_{65}$-*co*-DMA$_{214}$)-TTC; SEC: M_n = 43.6 kDa, $Đ$ = 1.28; denoted as **P3**. Another statistical copolymer was synthesized at HisAm/ DMA/PDMA$_{76}$-TTC/SPTP = 100:200: 1:0.25 under visible light for 8 min. ^{1}H NMR: 94% DMA, 95% HisAm conv. Weight: 1.832 g, Yield: 97%. ^{1}H NMR: PDMA$_{76}$-*b*-P(HisAm$_{95}$-*co*-DMA$_{188}$)-TTC; SEC: M_n = 45.3 kDa, $Đ$ = 1.25; denoted as **P2**. The yields for the synthesis of the statistical block imidazolium-copolymers were calculated using the same method described above. Unlike **P1** PHisAm homoblock, substantial DMA units were inserted in **P2** and **P3** PHisAm segments. This was termed as "substantial DMA-dilution" for macromolecular crowding and confinement.

Potentiometric Titration. **P1** (65.3 mg, 240 μmol units) was dissolved in water (2.0 mL). The solution was then adjusted close to pH 1.0 using 3.0 M HCl, titrated using 0.25 M NaOH. Unless otherwise mentioned, **P2**, **P3**, L-AspAm monomer, and the monomer/polymer coacervates were titrated using the same protocol.

Spontaneous LLPS Droplet Compartmentalization of Imidazolium-Copolymers in Water. **P1** (0.100 g, 3.68 μmol polymer) was dissolved in water (20.0 g) at pH 2.5. A 2.0-mL aliquot of the stock solution was transferred to a 10 mL vial and adjusted to pH 2.5, 3.2, 4.0, 5.0, 6.2, 7.0, and 7.3, followed by stirring at room temperature overnight. The resulting dispersion was analyzed using ^{1}H NMR, zeta potential, DLS, and TEM. The dispersions of **P2**, **P3** (5 mg/mL in water) were prepared and examined under the same conditions.

Site-Specific Monomer Recruitment/Localization into Droplet Compartments. Typically, **P1** (1.091 g, 40 μmol polymer) was dissolved in water (20.0 g) at pH 3.2, and stirred at room temperature overnight. Subsequently, L-AspAm monomer (0.374 g, 2.0 mmol) was added to the flask. The mixture was adjusted to pH 3.2 and stirred for 2 h, resulting in the stock solution at a monomer-to-polymer molar ratio (φ) of 50:1. A 2.0-mL aliquot of stock solution was transferred to a 20-mL vial, diluted to 9.1 mg/mL solids (2.3 mg/mL L-AspAm), and adjusted to pH 2.5, 3.2, 5.0, 6.2, and 7.0 respectively. The mixture was stirred overnight. The resulting dispersion was studied using potentiometric titration, ^{1}H NMR, zeta potential, DLS, TEM, and XRD. The impact of the composition (φ) on site-specific monomer recruitment into the *dense-phase* compartments of **P1**, **P2**, **P3** droplets at φ = 30, 50, 100, 150, 200, 300 in water at pH 3.2 were studied.

Kinetic Properties of Catalytic Coacervates for Photo-RAFT in Dilution. The protocol was as follows. *(1) Monomer Recruitment:* **P1** (1.091 g, 40 μmol polymer) was dissolved in water (20.0 g) in a flask. L-AspAm monomer (0.374 g, 2.0 mmol) was added to the flask. The mixture was adjusted to pH 3.2, and stirred at room temperature overnight. *(2) Heterogeneous Polymerization:* An aliquot of stock solution (1.005 g; 93 μmol L-AspAm and 1.9 μmol polymer) was transferred to a 5-mL reaction flask. Subsequently, SPTP initiator (1.0% w/w in water; 15 mg, 0.47 μmol) was added to the flask in darkness. The flask was immersed in a water bath at 25 °C. After purged with argon gas for 45 min, the flask was exposure to visible light to target time point. The reaction was quenched by exposure to air, addition of hydroquinone inhibitor. The kinetic properties of **P2** and **P3** monomer complex coacervates were studied under the above conditions. The dispersion was analyzed using potentiometric titration, ^{1}H NMR, zeta potential, DLS, TEM, and XRD. **K1**, **K2**, and **K3** denote the photo-RAFT reaction kinetics of the **P1**, **P2**, and **P3** monomer complex coacervates respectively.

As a control experiment, we studied kinetics of the L-AspAm polymerization using a small CTCPA chain transfer agent (in the absence of the polymer droplets) at L-AspAm/ CTCPA/SPTP = 50:1:0.25 in dilution at 0.5 M, 0.2 M, 0.1 M L-AspAm, respectively in water at pH 3.2, using the same procedures described above.

Instrumentation and Characterization

Potentiometric titration was performed using a FE28 digital pH meter with a LE422 electrode. The dialyzed polymer solution, the dispersions of monomer/polymer complex coacervates and the final reaction products were freeze-dried in a Labconco FreeZone 2.5 L freeze-drier. Incident visible light employed for aqueous photo-RAFT polymerization was obtained from a photo-synthesis system with a 400 W mercury lamp (UV light filtered using JB400 filters), a UV-A radiometer, and a ventilator.

^{1}H NMR spectra were recorded on a Bruker 400 MHz NMR spectrometer. *SEC analysis* was performed on a PL-GPC220 system with a refractive index detector, a column set (2 × PLGel MIXED-B + 1 × PLGel MIXED-D). The analysis used 10 mM LiBr containing DMF eluent and PMMA standards (Agilent, 1.43−1441 kDa). The calibration and analysis were conducted at a flow rate of 1.0 mL/min at 80 °C. To prevent adsorption onto GPC column, polymeric imidazole motifs were protected by reaction with di-*tert*-butylpyrocarbonate in methanol at pH 12 at 25 °C for 2 days. The solvents were removed under reduced pressure. The polymer was dissolved in $CHCl_3$ and passed through a silica column. After rotary evaporation, the polymer sample was dissolved in DMF. Prior to the SEC analysis, the solution was filtered using a 0.20 μm filter.

Particle ζ-potential was measured using a Malvern Zetasizer Nano-ZS90 instrument, with data averaged over five runs. *DLS analysis* was performed on a Brookhaven BI-200SM setup with a 22-mW He-Ne laser (λ = 633 nm), a BI-200SM goniometer, and a BI-Turbo Corr digital correlator.

The sample cell was maintained at 25 °C using a BI-TCD temperature controller, measurements were taken at an angle of 90°. Data were recorded through cumulants analysis utilizing CONTIN routine and averaged over five runs. *TEM characterization* was conducted on a Hitachi HT7700 transmission electron microscope at an accelerating voltage of 120 kV. *Cryofixation method* was employed for TEM sample preparation: an aliquot of the dispersion (10 μL) was placed onto a carbon-coated copper grid. The liquid sample underwent cryofixation by immersion in liquid nitrogen for 15 min, after which it was transferred to a custom-made cryobox for an additional 2 hours of freezing in liquid nitrogen. The sample was lyophilized in vacuum overnight. Prior to TEM analysis, freeze-dried sample was further dried in a P_2O_5-drying desiccator. XRD analysis was conducted using a Bruker D8 ADVANCE diffractometer equipped with a Cu Kα X-ray source at $\lambda = 1.5418$ Å. The domain spacing (*d*-spacing) was calculated based on Bragg equation $d = \lambda/(2 \times \sin\theta)$, in which λ and θ denote X-ray wavelength and scattering angle, respectively.

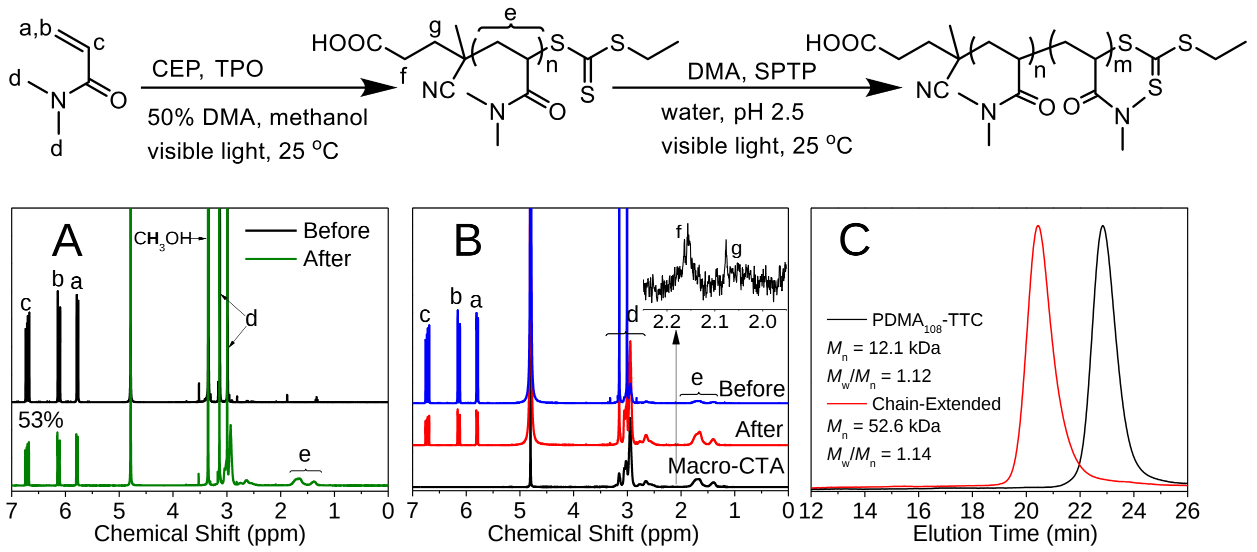

Figure S1. (A) ^{1}H NMR spectra of reaction solutions before/after RAFT polymerization of DMA monomer using CEP chain transfer agent and TPO initiator at DMA/CEP/TPO =200:1:0.35 at 50% w/w DMA in methanol under visible light at 25 °C for 45 min, conversion was determined using Eq. S3; (B) those before and after chain extension of as-synthesized $PDMA_{108}$-TTC macro-CTA at DMA/$PDMA_{108}$-TTC/SPTP=500:1:0.25 at 20% w/w total solids in water at pH 2.5 under visible light at 25 °C for 3 min; the degree of polymerization (DP, n = 108) of macro-CTA was determined using Eq. S4, I_{f+g} denotes integral signals *f*, *g* of CEP end-groups (*inset*). (C) DMF SEC traces of $PDMA_{108}$-TTC (*black*) and the chain-extended copolymer (*red*).

$$Conversion = \left(1 - \frac{I_a^t/I_d^t}{I_a^0/I_d^0}\right) \times 100\% \qquad \text{(S3)}$$

$$DP = \frac{2\times I_d}{3\times I_{f+g}} \qquad \text{(S4)}$$

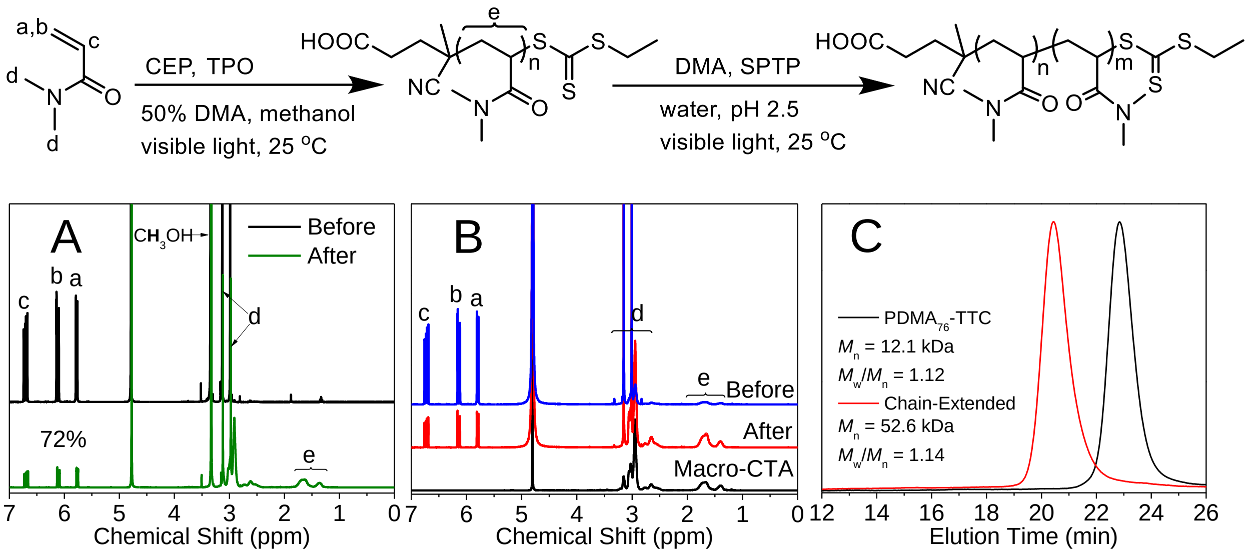

Figure S2. (A) ^{1}H NMR spectra of solutions before/after RAFT polymerization of DMA at DMA/CEP/TPO = 100:1:0.35 at 50% w/w DMA in methanol, under visible light at 25 °C for 200 min; (B) those before and after chain extension of above-synthesized PDMA$_{76}$-TTC macro-CTA (DP = 76) at DMA/PDMA$_{76}$-TTC/SPTP = 500:1:0.25 at 20% w/w solids in water at pH 2.5 under visible light at 25 °C for 3 min. (C) DMF SEC traces of the PDMA$_{76}$-TTC macro-CTA (*black*) and chain-extended copolymer (*red*). Conversion and DP (n) were determined using Eq. S3, S4.

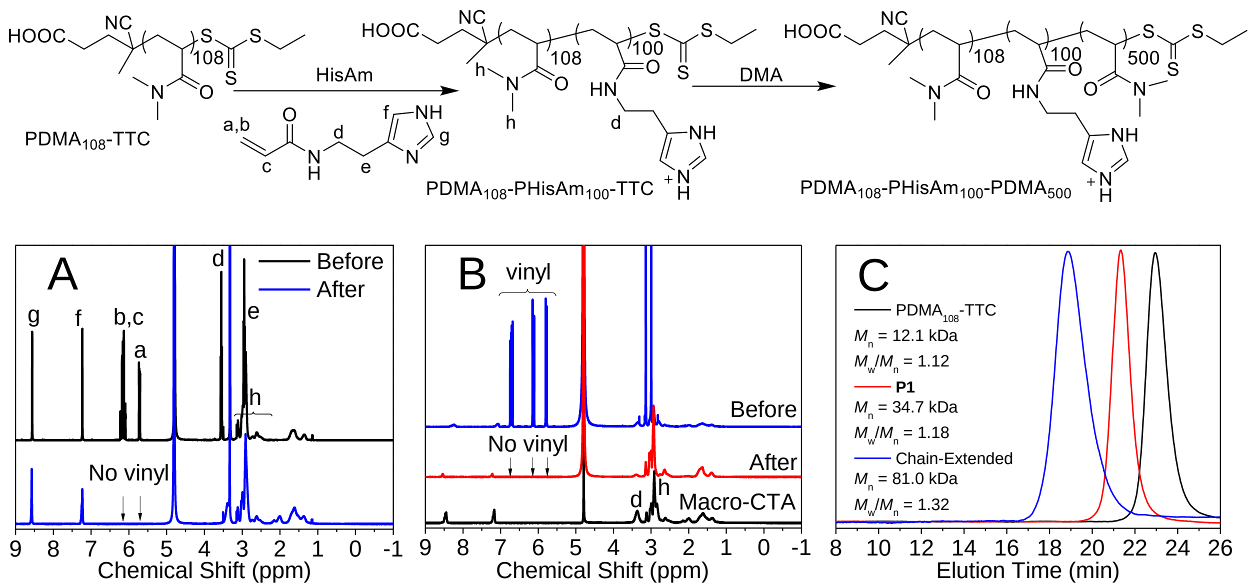

Figure S3. (A) ^{1}H NMR spectra of solutions before/after RAFT block copolymerization of HisAm monomer using $PDMA_{108}$-TTC macro-CTA at HisAm/$PDMA_{108}$-TTC/SPTP = 100:1:0.35 at 40% w/w solids in 3/7 w/w methanol/water at pH 2.5, under visible light at 25 °C for 20 min (*arrow*: 100% conv.); (B) those before and after chain extension of $PDMA_{108}$-*b*-$PHisAm_{100}$-TTC macro-CTA (**P1**) at DMA/ TTC/SPTP = 500:1:0.25 at 20% w/w solids in water at pH 2.5, under visible light at 25 °C for 15 min, in which DP_{PHisAm} was determined based on DP_{PDMA} = 108 using Eq. S5, I_d and I_h are integral signals *d* (HisAm), *h* (DMA unit). (C) SEC traces of $PDMA_{108}$-TTC (*black*), $PDMA_{108}$-*b*-$PHisAm_{100}$-TTC (**P1**, *red*), and the chain-extended copolymer (*blue*).

$$DP_{PHisAm} = \frac{3 \times I_d \times DP_{PDMA}}{I_h} \qquad \text{(S5)}$$

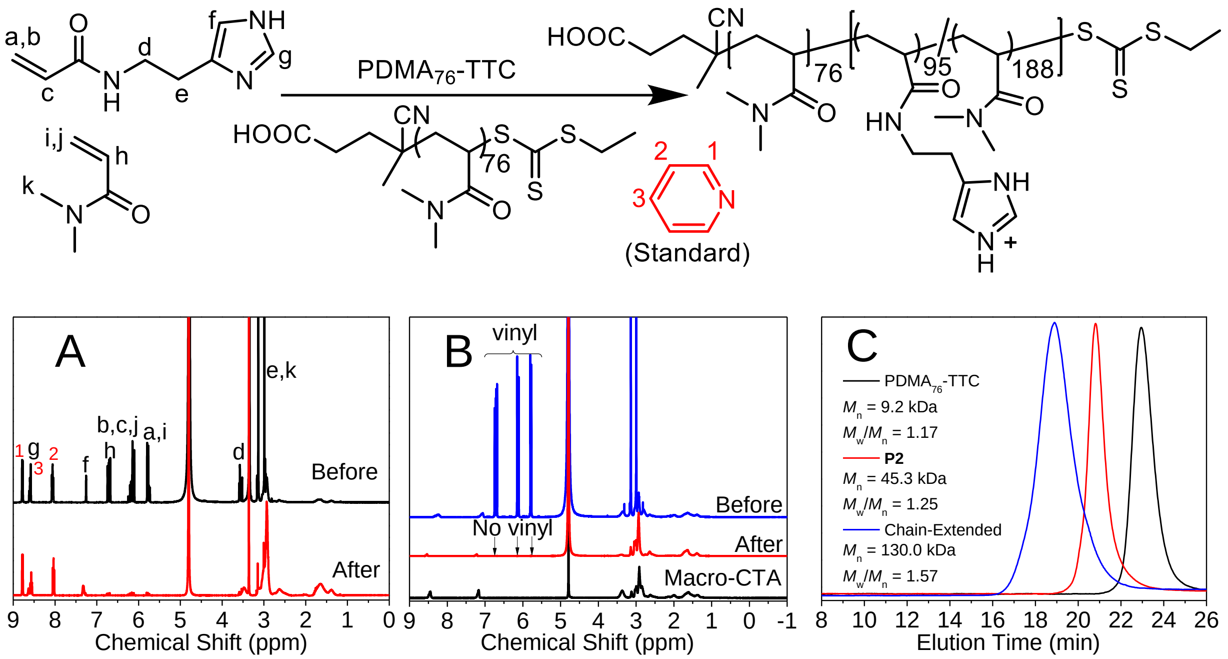

Figure S4. (A) ^{1}H NMR spectra of solutions before/after RAFT copolymerization of HisAm and DMA using PDMA76-TTC macro-CTA at HisAm/DMA/PDMA76-TTC/SPTP = 100:200:1:0.25 at 40% w/w solids in 3/7 w/w methanol/water at pH 2.5 under visible light at 25 °C for 8 min, leading to 94% DMA and 95% HisAm conv. as determined by ^{1}H NMR using Eq. S6 and S7, in which I_{a+i}, I_h and I_1 are integral signals of *a* (HisAm), *i* (DMA), *h* (DMA), and *1* (pyridine), the polymer composition was evaluated based on conversions; (B) those before/after chain extension of as-achieved PDMA76-*b*-P(HisAm95-*co*-DMA188)-TTC (**P2**) at DMA/TTC/SPTP = 500:1:0.25 at 20% w/w solids at pH 2.5, under visible light at 25 °C for 15 min. (C) SEC traces of PDMA76-TTC (*black*), PDMA76-*b*-P(HisAm95-*co*-DMA188)-TTC (**P2**, *red*) and chain-extended copolymer (*blue*).

$$Conversion_{DMA} = \left(1 - \frac{I_h^t/I_1^t}{I_h^0/I_1^0}\right) \times 100\% \qquad \text{(S6)}$$

$$Conversion_{HisAm} = \left(1 - \frac{(I_{a+i}^t - I_h^t)/I_1^t}{(I_{a+i}^0 - I_h^0)/I_1^0}\right) \times 100\% \qquad \text{(S7)}$$

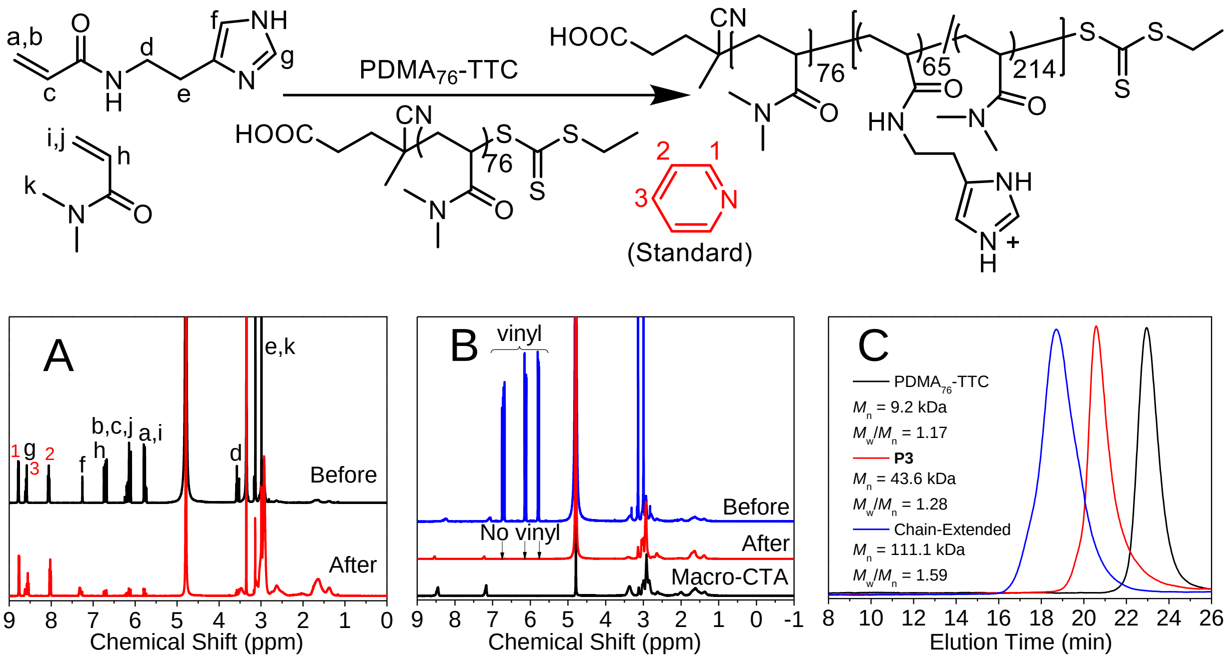

Figure S5. (A) ^{1}H NMR spectra of solutions before/after RAFT copolymerization of HisAm/DMA monomers at HisAm/DMA/PDMA$_{76}$-TTC/SPTP = 70:230:1:0.25 at 40% w/w solids in 3/7 w/w methanol/water at pH 2.5, under visible light at 25 °C for 6 min up to 93% DMA and 93% HisAm conv., determined using Eq. S6, S7; (B) those before/after the chain extension of as-synthesized PDMA$_{76}$-*b*-P(HisAm$_{65}$-*co*-DMA$_{214}$)-TTC (**P3**) at DMA/TTC/SPTP = 500:1:0.25 at 20% w/w solids at pH 2.5, under visible light at 25 °C for 15 min. (C) SEC traces of PDMA$_{76}$-TTC (*black*), PDMA$_{76}$-*b*-P(HisAm$_{65}$-*co*-DMA$_{214}$)-TTC (**P3**, *red*) and the chain-extended copolymer (*blue*).

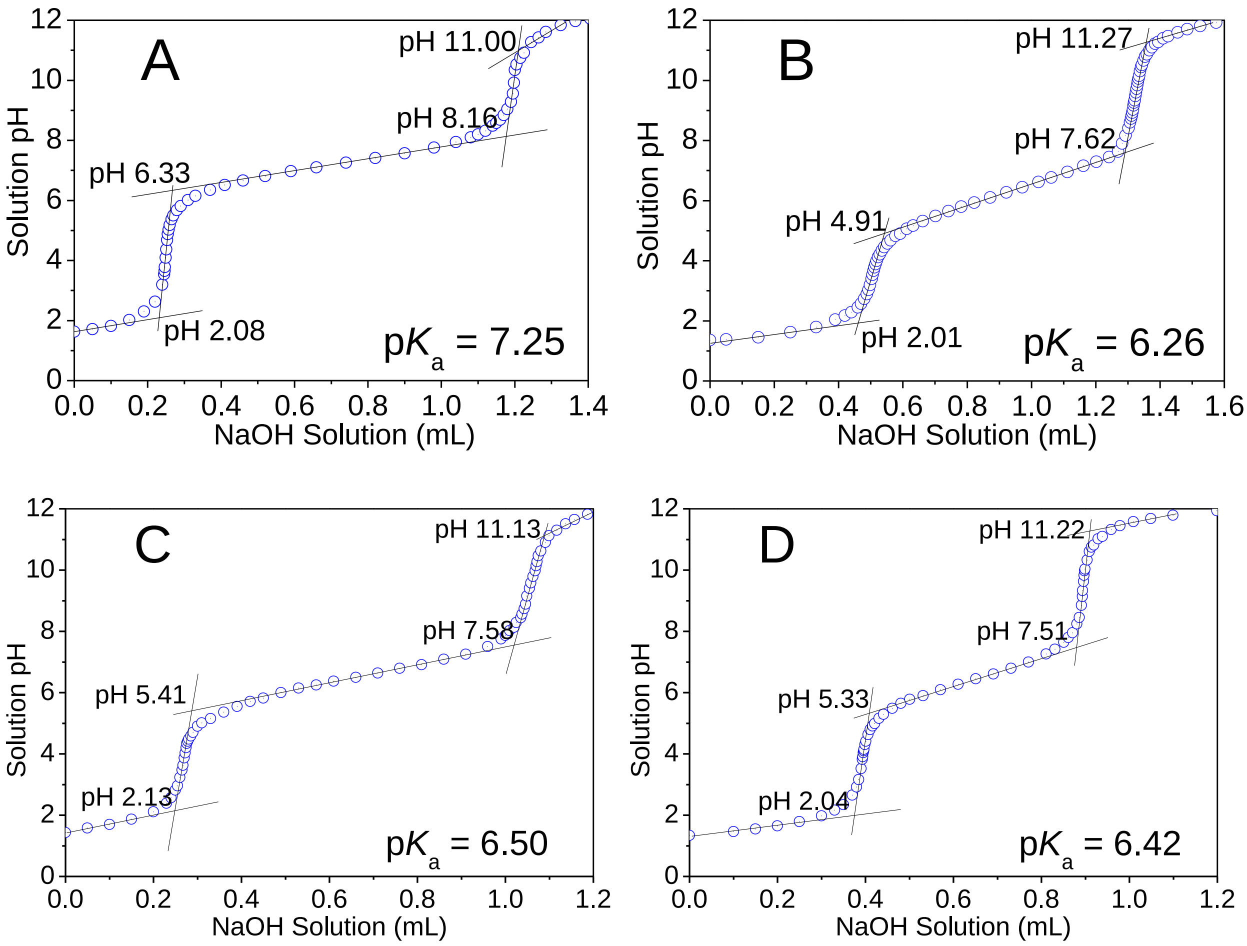

Figure S6. Titration plots of (A) HisAm monomer, (B) **P1** ($PDMA_{108}$-*b*-$PHisAm_{100}$-TTC), (C) **P2** ($PDMA_{76}$-*b*-P($HisAm_{95}$-*co*-DMA_{188})-TTC), (D) **P3** ($PDMA_{76}$-*b*-P($HisAm_{65}$-*co*-DMA_{214})-TTC) at 0.12 M HisAm monomer/units in water using a 0.25 M NaOH titrant.

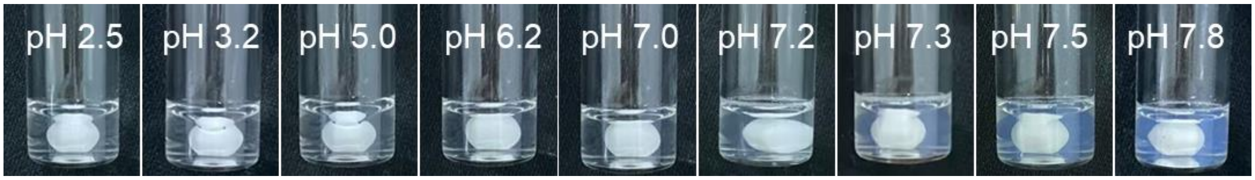

Figure S7. Digital photographs of the **P1** dispersions at 5.0 mg/mL in water at labeled pH values.

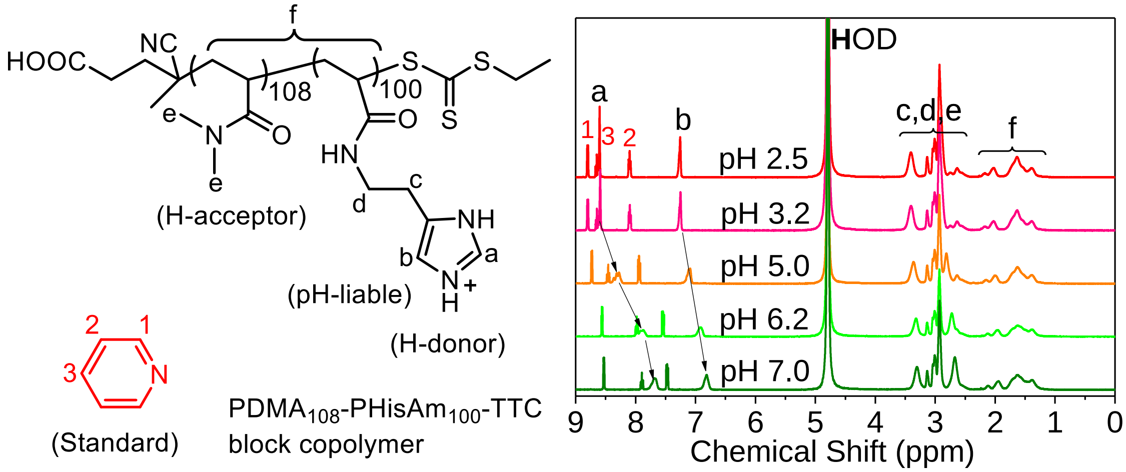

Figure S8. ^{1}H NMR spectra of **P1** recorded at 6.0 mg/mL in D_2O at labeled pH values. Pyridine (pyridine/HisAm unit = 1:4 mol/mol) was used to determine PHisAm (γ_1), PDMA (γ_2) dehydration using Eq. S8, S9. The results indicate complete segment hydration in both imidazole ionization and deionization states at pH 2.5–7.0.

$$\gamma_1 = \left(1 - \frac{I_b}{2\times I_1}\right) \times 100\% \qquad \text{(S8)}$$

$$\gamma_2 = \left(1 - \frac{I_{c+d+e} - 4\times I_b}{13\times I_1}\right) \times 100\% \qquad \text{(S9)}$$

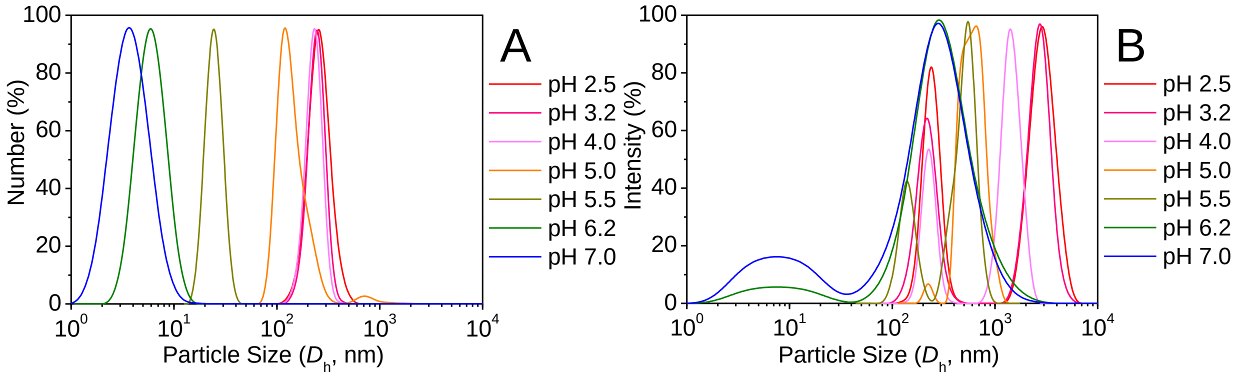

Figure S9. (A) Number- and (B) intensity-average **P1** particle size distribution (D_h: hydrodynamic diameter) at 5.0 mg/mL in water at labeled pH values.

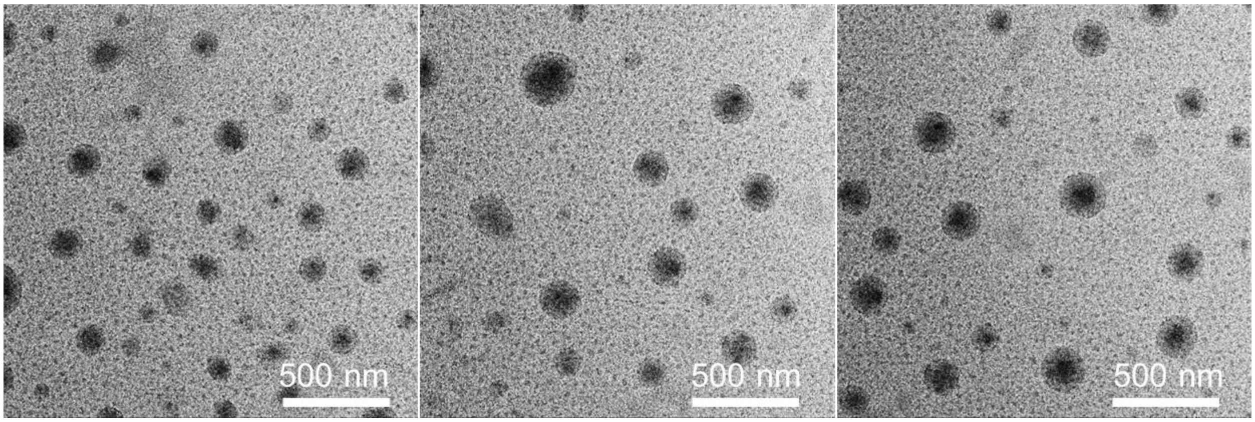

Figure S10. Cryofixation TEM images of the **P1** droplets (small dots: nascent clusters) at pH 2.5.

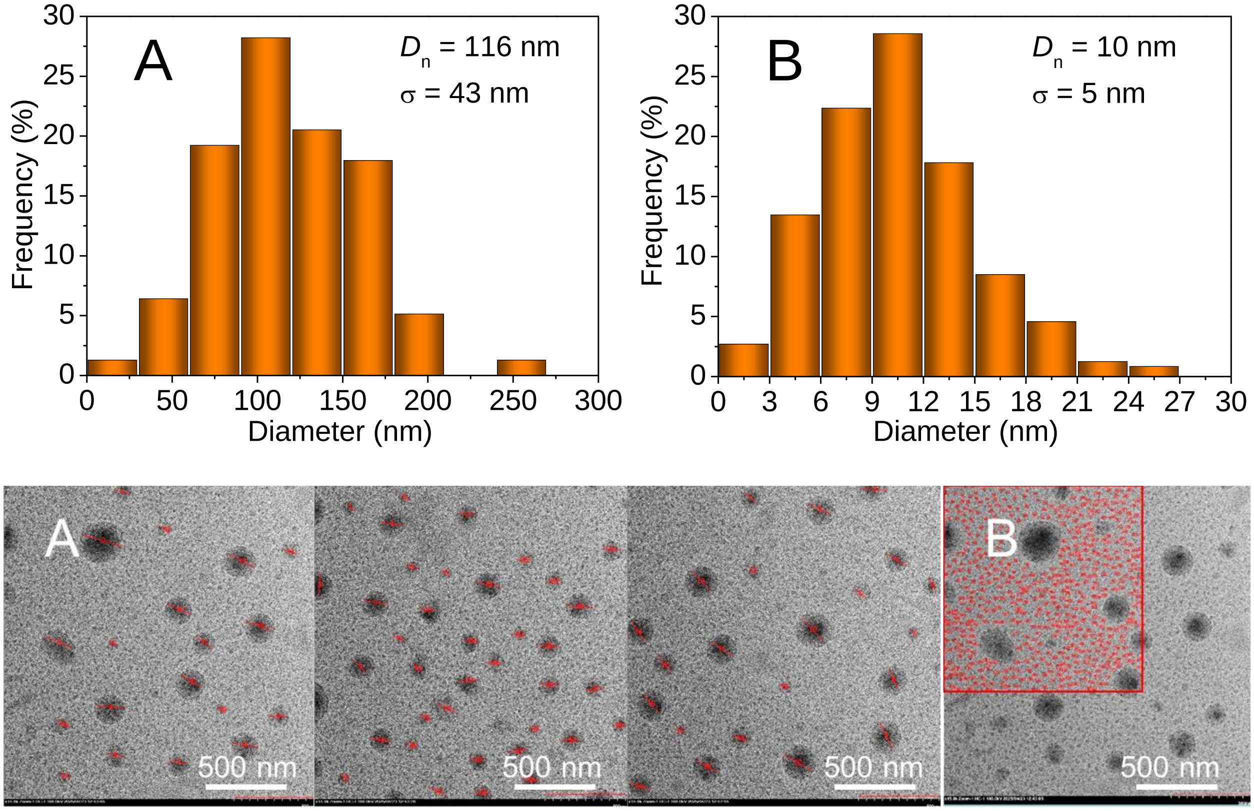

Figure S11. Cryofixation TEM statistical analysis data (D_n: mean diameter, σ: standard deviation) of **P1** droplets (A) and small dots (nascent clusters, B) in water at pH 2.5.

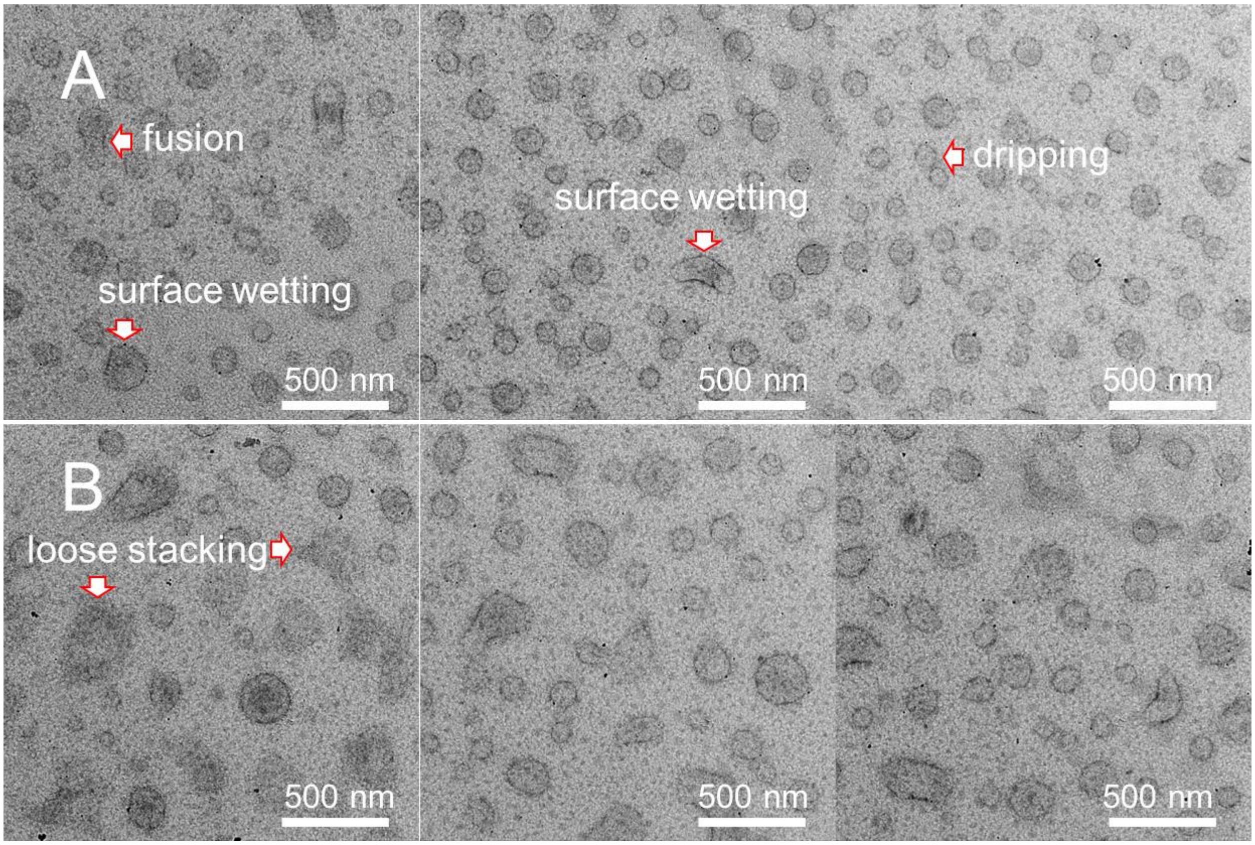

Figure S12. Cryofixation TEM images of (A) **P2**, (B) **P3** droplets and the nascent clusters at pH 2.5. Here, the deformed bubbles (*arrows*) indicate intrinsic droplet liquid fluidity (P. Dogra, et al. J. Am. Chem. Soc. 2019, 141, 20380−20389). Those ambiguous images indicate the loose stacking of nascent clusters leading to the newly forming droplets *via* hierarchical ICC-driven LLPS droplet compartmentalization.

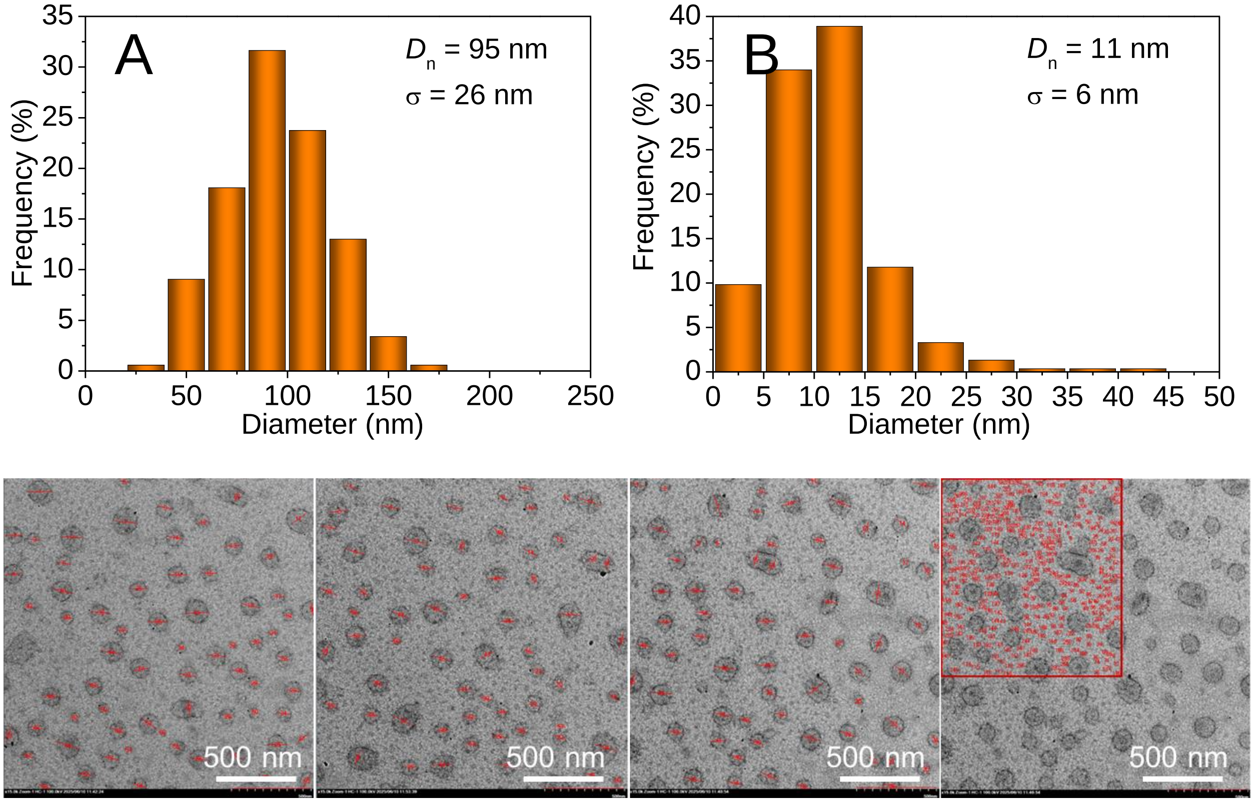

Figure S13. TEM statistical analysis data (D_n: mean diameter, σ: standard deviation) of (A) **P2** droplets and (B) nascent clusters at pH 2.5.

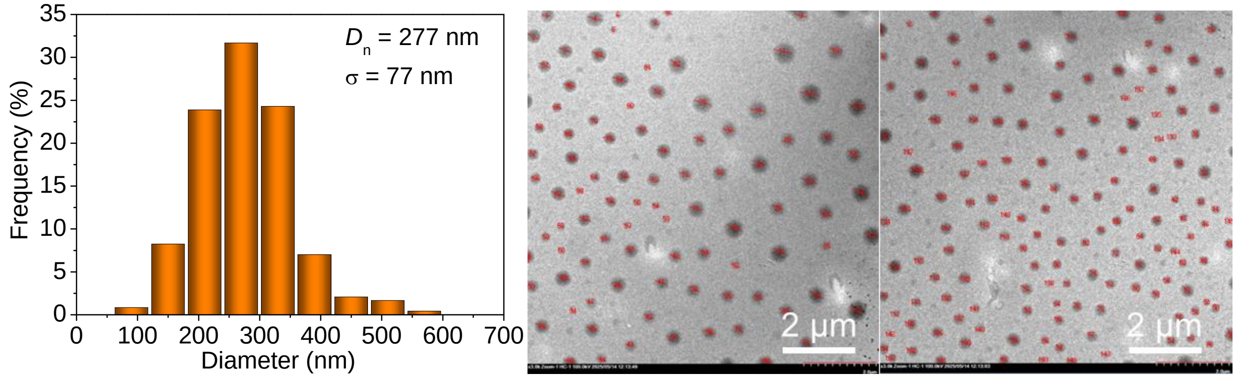

Figure S14. TEM statistical analysis data (D_n: mean diameter, σ: standard deviation) of the **P2** uniform droplets at pH 5.0.

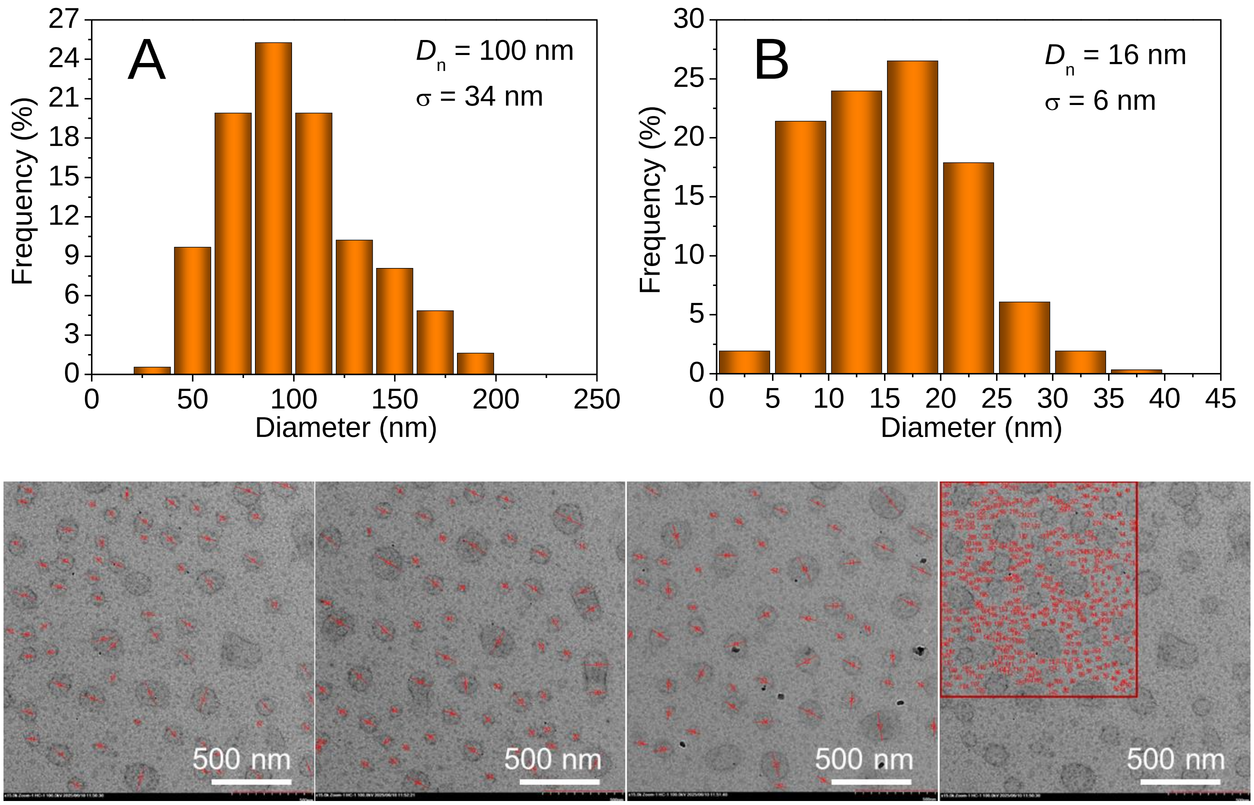

Figure S15. TEM statistical analysis data (D_n: mean diameter, σ: standard deviation) of the (A) **P3** droplets and (B) nascent clusters at pH 2.5.

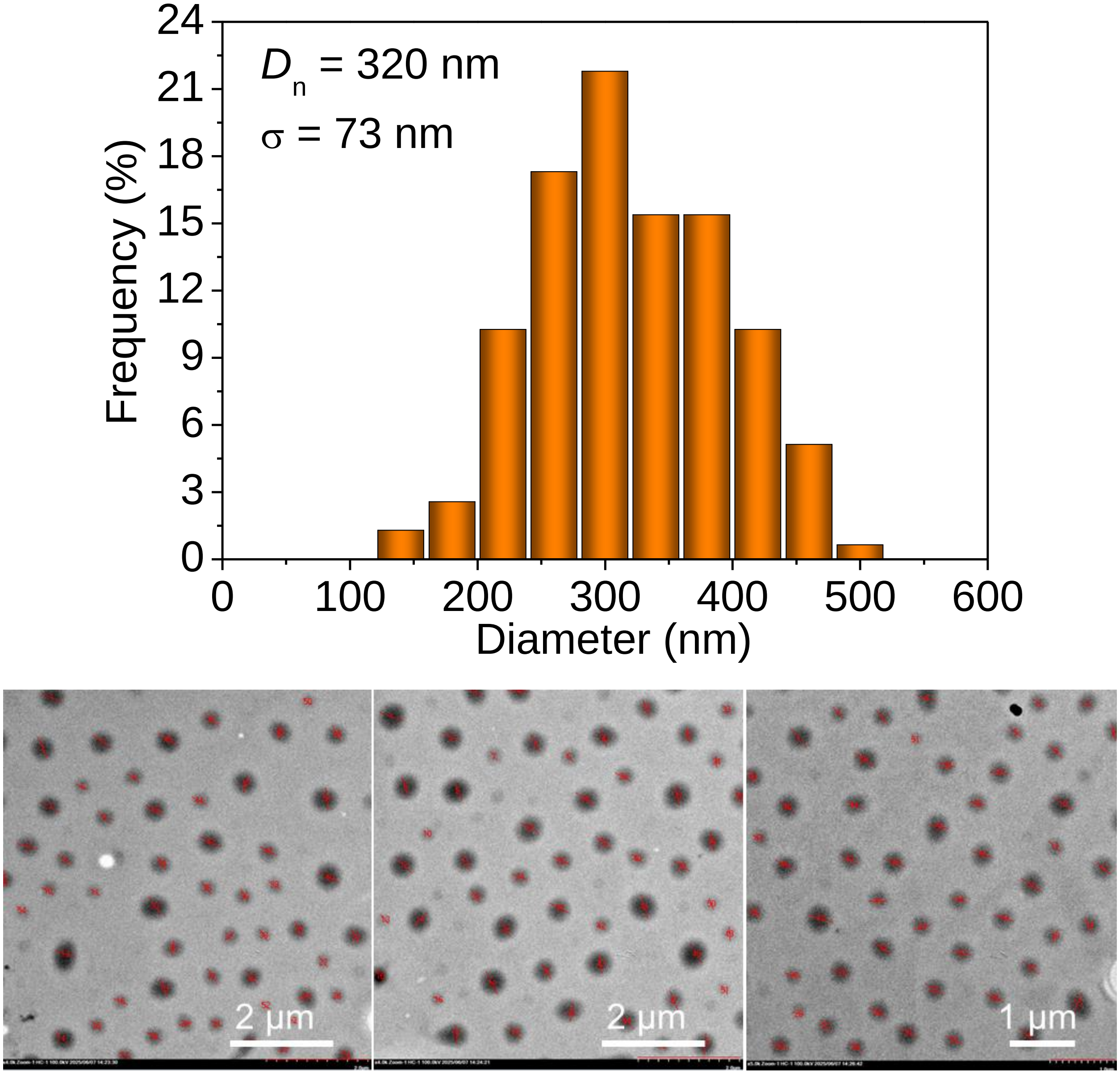

Figure S16. TEM statistical analysis data (D_n: mean diameter, σ: standard deviation) of the **P3** uniform droplets at pH 5.0.

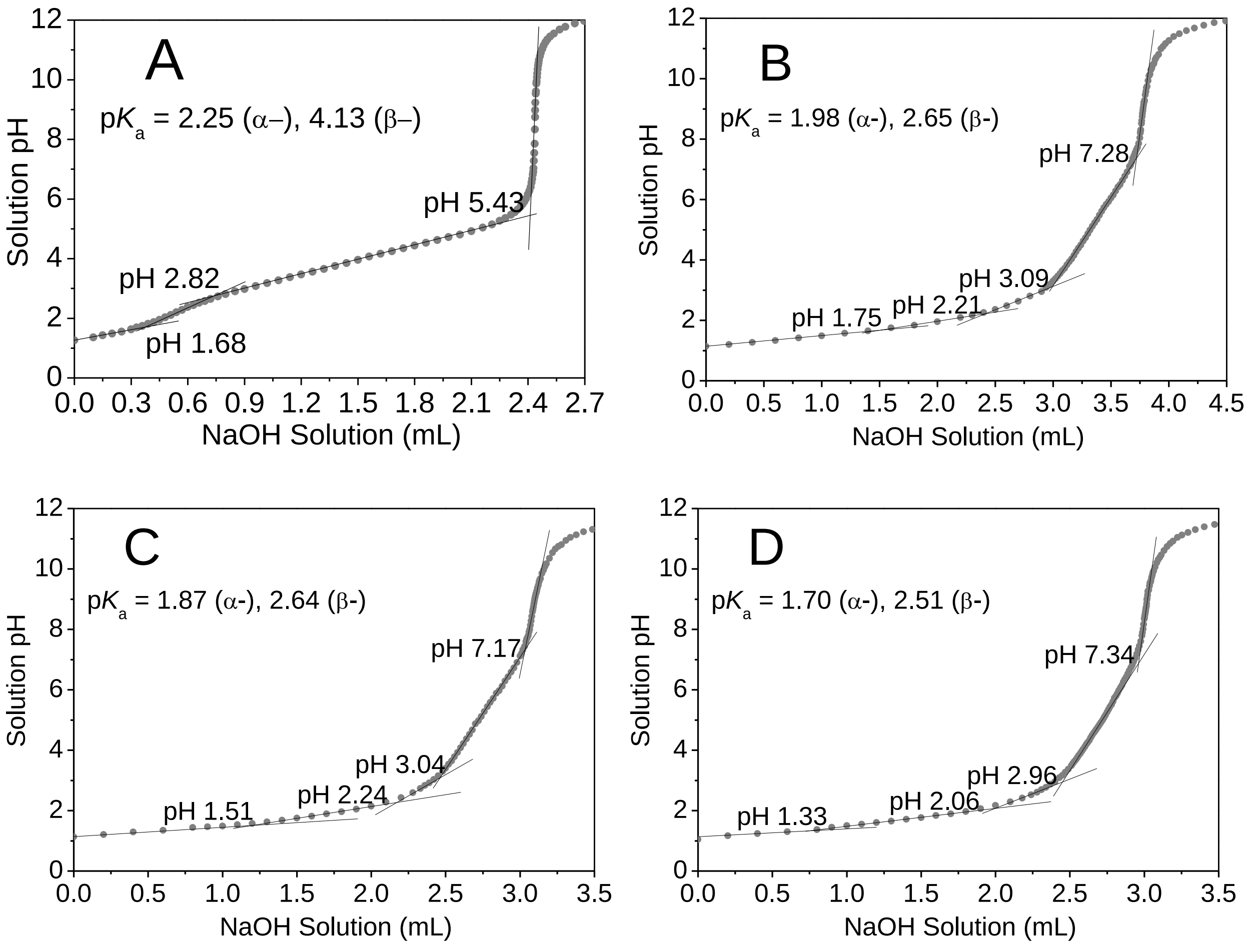

Figure S17. Titration plots of (A) 0.10 M L-AspAm (2.0 mL, titrated with 0.20 M NaOH), and monomer complex coacervates of (B) **P1**/(C) **P2**/(D) **P3** droplets at monomer/polymer (φ) = 50:1 (molar ratio) at 30 mM (AspAm + HisAm units) in water, titrated with 65 mM NaOH.

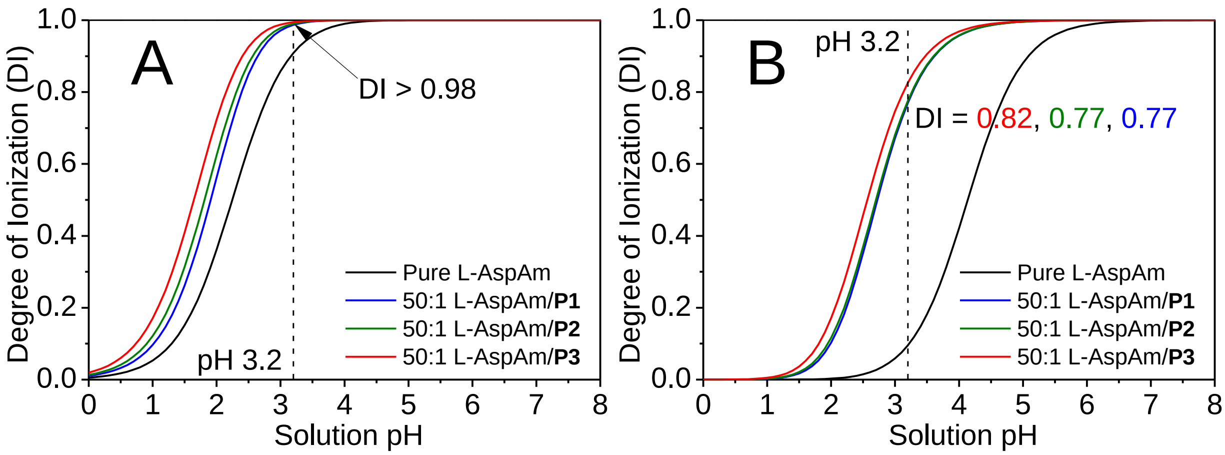

Figure S18. Degrees of ionization (DI) of (A) α-COOH, (B) β-COOH in pure L-AspAm (*black*), and the monomer complex coacervates with **P1** (*blue*), **P2** (*green*), **P3** (*red*), determined using Eq. S10 and S11, in which subscript 1 and 2 denote α- and β-COOH in L-AspAm monomer.

$$DI_1 = \left(1 - \frac{\left(10^{-pH}\right)^2}{(10^{-pH})^2 + 10^{(-pK_{a,1}-pH)} + 10^{(-pK_{a,1}-pK_{a,2})}}\right) \times 100\% \qquad \text{(S10)}$$

$$DI_2 = \frac{10^{(-pK_{a,1}-pK_{a,2})}}{(10^{-pH})^2 + 10^{(-pK_{a,1}-pH)} + 10^{(-pK_{a,1}-pK_{a,2})}} \times 100\% \qquad \text{(S11)}$$

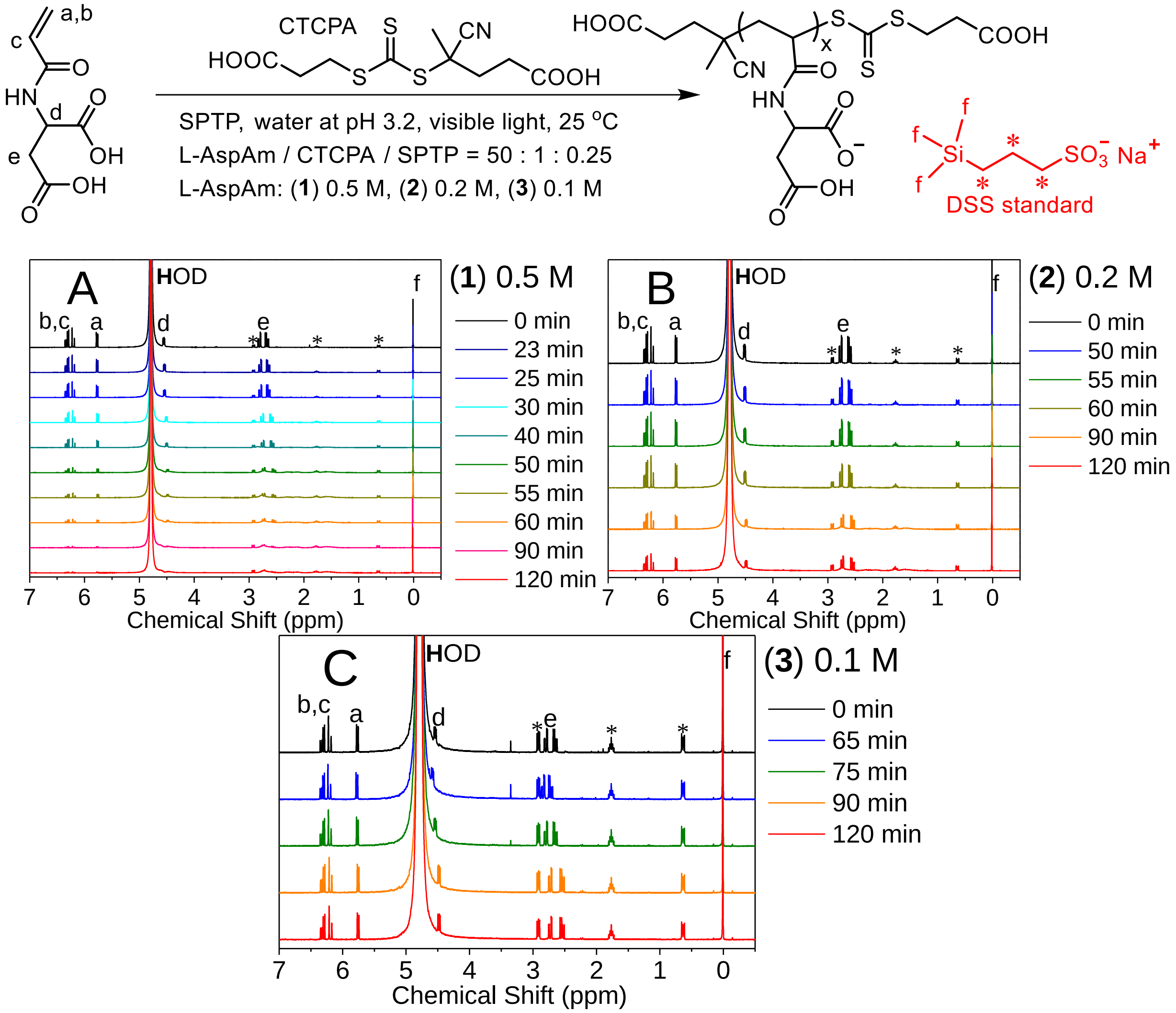

Figure S19. (A) ^{1}H NMR spectra (recorded in D_2O at pH 9.0) of reaction solutions at labeled time points of aqueous solution photo-RAFT polymerization of L-AspAm using CTCPA chain transfer agent and SPTP initiator at L-AspAm/CTCPA/SPTP = 50:1:0.25 at (A) 0.5 M (**1**), (B) 0.2 M (**2**), (C) 0.1 M AspAm (**3**) in water at pH 3.2, under visible light at 25 °C. Conversion was determined by comparing vinyl signal *a* with DSS signal *f*, using Eq. S12.

$$Conversion = \left(\frac{I_a^0/I_f^0 - I_a^t/I_f^t}{I_a^0/I_f^0}\right) \times 100\% \qquad \text{(S12)}$$

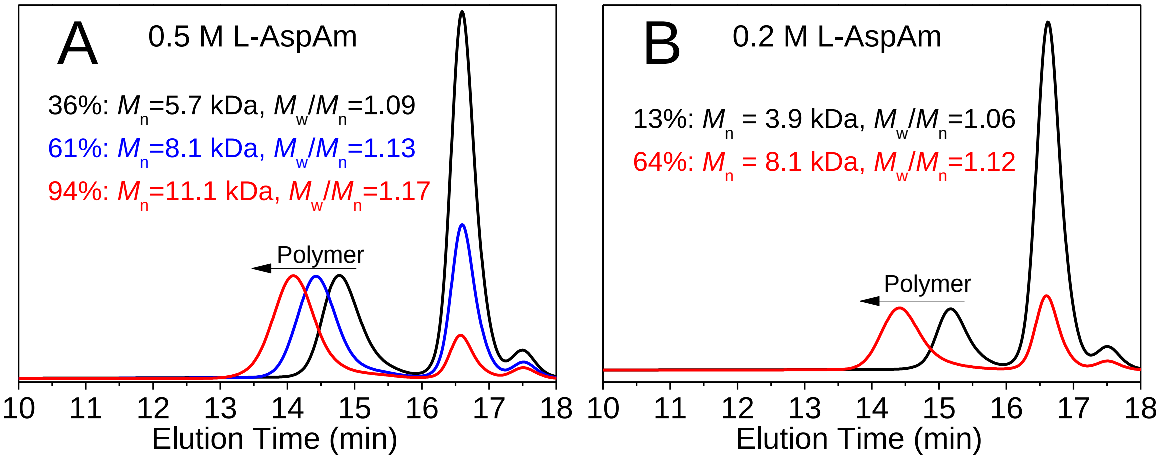

Figure S20. Aqueous SEC traces of reaction mixtures at labeled conversions of the polymerization at L-AspAm/CTCPA/SPTP = 50:1:0.25 at (A) 0.5 M, (B) 0.2 M AspAm in water at pH 3.2, under visible light at 25 °C.

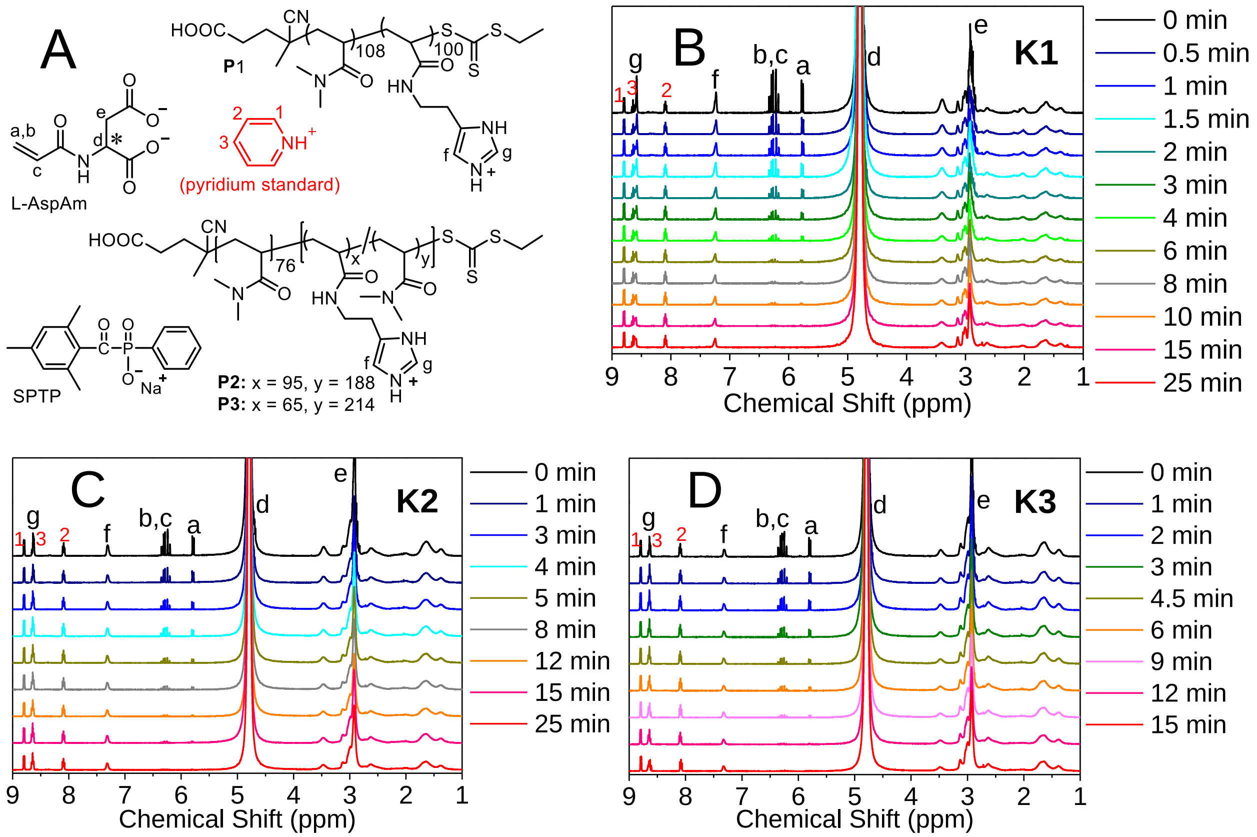

Figure S21. (A) Molecular structures of **P1**, **P2**, **P3**, L-AspAm monomer, and SPTP initiator used for heterogeneous photo-RAFT polymerizations in dilution named as **K1**, **K2** and **K3** respectively. ^{1}H NMR spectra of (B) **K1**, (C) **K2**, (D) **K3** reaction coacervates at labeled time points of photo-RAFT using SPTP initiator at L-AspAm/TTC/SPTP = 50:1:0.25 (TTC: polymeric trithiocarbonate end-groups) in the dilution at 1.8% w/w (0.10 M) L-AspAm in water at pH 3.2, under visible light at 25 °C. Conversion was determined comparing vinyl signals *b*, *c* with added cationic pyridinium signal 1 according to Eq. S13.

$$Conversion = \left(1 - \frac{I_{b,c}^{t}/I_{1}^{t}}{I_{b,c}^{0}/I_{1}^{0}}\right) \times 100\% \qquad \text{(S13)}$$

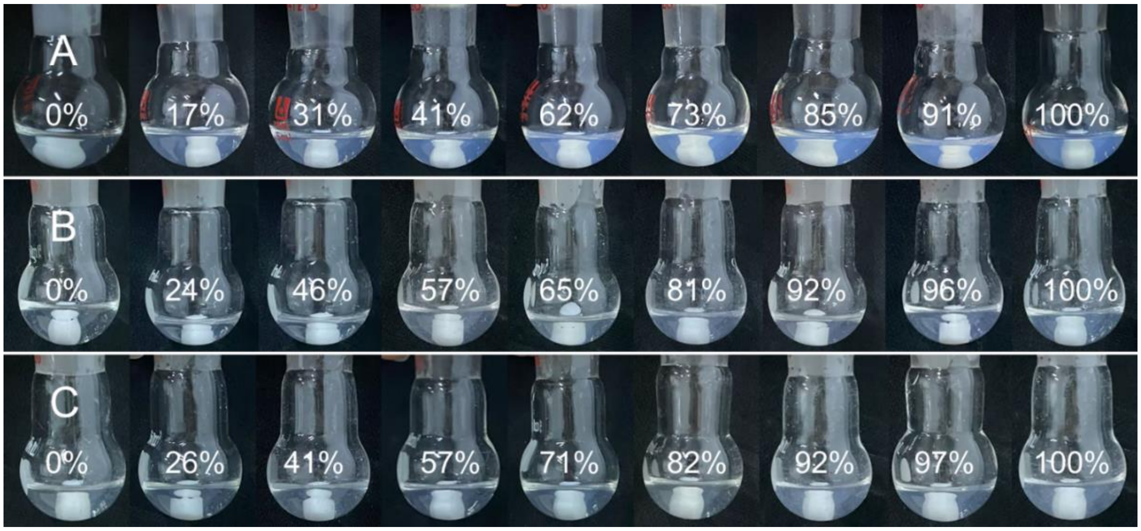

Figure S22. Digital photographs of (A) **K1**, (B) **K2**, and (C) **K3** dispersions at labeled conversions.

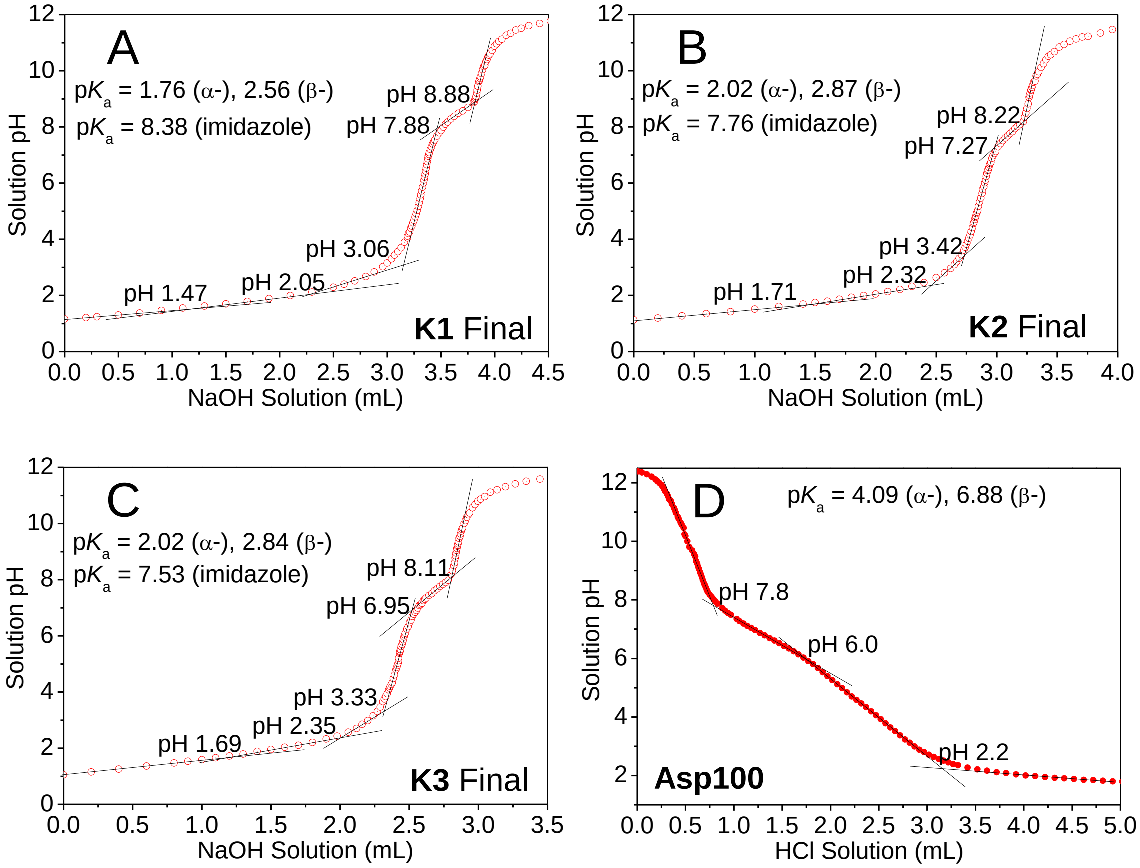

Figure S23. Titration plots of (A) **K1**, (B) **K2**, (C) **K3** final products and (D) $PAspAm_{100}$ (**Asp100**).

Reference

1. Thang, S.H.; Chong, Y.K.; Mayadunne, R.T.A.; Moad, G.; Rizzardo, E. A novel synthesis of functional dithioesters, Dithiocarbamates, xanthates and trithiocarbonates. *Tetrahedron Lett.* **1999**, *40*, 2435−2438.
2. Luo, C.; Wang, X.; Liu, Y.; Cai, J.; Lu, X.; Cai, Y. Like-charge PISA: Polymerization-induced like-charge electrostatic self-assembly. *ACS Macro Lett.* **2023**, *12*, 1045−1051.
3. Cao, L.; Zhao, Q.; Liu, Q.; Ma, L.; Li, C.; Wang, X.; Cai, Y. Electrostatic manipulation of triblock terpolymer nanofilm compartmentalization during aqueous photoinitiated polymerization-induced self-assembly. *Macromolecules* **2020**, *53*, 2220−2227.
4. Bronstert, B.; Henne, A.; Hesse, A.; Jacobi, M.; Wallbillich, G. "Acylphosphine compounds and their use as photoinitiators." U.S. Patent 4719297, 1988.
5. Shi, Y.; Liu, G.; Gao, H.; Lu, L.; Cai, Y. Effect of mild visible light on rapid aqueous RAFT polymerization of water-soluble acrylic monomers at ambient temperature: Initiation and activation. *Macromolecules* **2009**, *42*, 3917−3926.
